# The genetic duet of concurrent *RASAL1* and *PTEN* alterations promotes cancer aggressiveness by cooperatively activating the PI3K–AKT pathway

**DOI:** 10.1002/1878-0261.13701

**Published:** 2024-07-20

**Authors:** Xiaopei Shen, Jie Tan, Rengyun Liu, Guangwu Zhu, Lisa Rooper, Mingzhao Xing

**Affiliations:** ^1^ Division of Endocrinology, Diabetes & Metabolism, Department of Medicine Johns Hopkins University School of Medicine Baltimore MD USA; ^2^ Department of Pathology Johns Hopkins University School of Medicine Baltimore MD USA

**Keywords:** cancer aggressiveness, PI3K pathway, *PTEN*, *RASAL1*, tumor suppressor gene

## Abstract

The significance of the prominent tumor suppressor gene for RAS protein activator‐like 1 (*RASAL1*) could be better understood by combined genetic, clinical, and functional studies. Here, we investigated the oncogenic and clinical impacts of genetic alterations of *RASAL1*, particularly when coexisting with genetic alterations of the gene for phosphatase and tensin homolog (*PTEN*), in 9924 cancers of 33 types in the TCGA database. We found common concurrent genetic alterations of the two genes, which were cooperatively associated with activation of the phosphatidylinositol 3‐kinase (PI3K)–AKT pathway, with cancer progression and mortality rates being 46.36% and 31.72% with concurrent gene alterations, versus 29.80% and 16.93% with neither gene alteration (HR 1.64, 95% CI 1.46–1.84 and 1.77, 95% CI 1.53–2.05), respectively. This was enhanced by additional tumor protein p53 (*TP53*) gene alterations, with cancer progression and mortality rates being 47.65% and 34.46% with coexisting *RASAL1*, *PTEN*, and *TP53* alterations versus 25.30% and 13.11% with no alteration (HR 2.21, 95% CI 1.92–2.56 and 2.76, 95% CI 2.31–3.30), respectively. In the case of breast cancer, this genetic trio was associated with a triple‐negative risk of 68.75% versus 3.83% with no genetic alteration (RR 17.94, 95% CI 9.60–33.51), consistent with the aggressive nature of triple‐negative breast cancer. Mice with double knockouts of *Rasal1* and *Pten* displayed robust Pi3k pathway activation, with the development of metastasizing malignancies, while single gene knockout resulted in only benign neoplasma. These results suggest that RASAL1, like PTEN, is a critical player in negatively regulating the PI3K–AKT pathway; defect in RASAL1 causes RAS activation, thus initiating the PI3K–AKT pathway signaling, which cannot terminate with concurrent PTEN defects. Thus, the unique concurrent *RASAL1* and *PTEN* defects drive oncogenesis and cancer aggressiveness by cooperatively activating the PI3K–AKT pathway. This represents a robust genetic mechanism to promote human cancer.

## Graphical abstract

Comprehensive analyses of TCGA revealed common coexisting *RASAL1* and *PTEN* alterations and their association with over‐activation of the PI3K–AKT pathway and poor clinical outcomes of cancer, which was recapitulated in mice with double *Rasal1–Pten* knockout and worsened by coexisting *TP53* alterations. Dual *Rasal1‐Pten* defects thus form a robust genetic mechanism to promote cancer aggressiveness through cooperatively activating the PI3K‐AKT pathway.

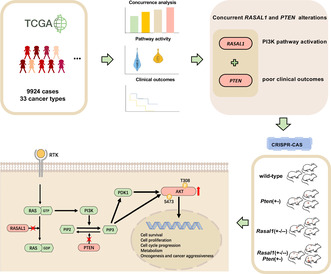

**Abbreviations**
ACC: adrenocortical carcinoma
AML: acute myeloid leukemia
ATC: anaplastic thyroid cancer
BLCA: bladder urothelial cancer
BRCA: breast invasive carcinoma
CESC: cervical squamous cell carcinoma and endocervical adenocarcinoma
CGC: cancer gene census
CHOL: cholangiocarcinoma
COAD: colon adenocarcinoma
CRISPR‐CAS 9: clustered regularly interspaced palindromic repeats‐associated protein 9
DLBC: diffuse large B‐cell lymphoma
DSS: disease‐specific survival
ER: estrogen receptor
ERK: extracellular signal‐regulated kinase
ESCA: esophageal carcinoma
FTC: follicular thyroid cancer
GBM: glioblastoma multiforme
GDP: guanosine diphosphate
GISTIC: genomic identification of significant targets in cancer
GTP: guanosine triphosphate
HER2: human epidermal growth factor receptor 2
HNSC: head and neck squamous cell carcinoma
HR: hazard ratio
KICH: kidney chromophobe
KIRC: kidney renal clear cell carcinoma
KIRP: kidney renal papillary cell carcinoma
KO: knockout
LGG: low‐grade glioma
LIHC: liver hepatocellular carcinoma
LUAD: lung adenocarcinoma
LUSC: lung squamous cell carcinoma
MAPK: the mitogen‐activated protein kinase
MESO: mesothelioma
MMRRC: mutant mouse resource & research centers
OV: ovarian serous cystadenocarcinoma
PAAD: pancreatic adenocarcinoma
PCPG: pheochromocytoma and paraganglioma
PFS: progression‐free survival
PI3K–AKT: phosphatidylinositol 3′‐kinase–AKT
PR: progesterone receptor
PRAD: prostate adenocarcinoma
PTC: papillary thyroid carcinoma
PTEN: phosphatase and tensin homolog
RASAL1: RAS protein activator like 1
RasGAP: RAS GTPase‐activating proteins
READ: rectal adenocarcinoma
RPPA: the reverse phase protein array
SARC: sarcoma
SKCM: cutaneous melanoma
STAD: stomach adenocarcinoma
TCGA: the cancer genome atlas
TGCT: testicular germ cell cancer
THCA: thyroid carcinoma
THYM: thymoma
TP53: tumor protein P53
UCEC: uterine corpus endometrial carcinoma
UCS: uterine carcinosarcoma
UVM: uveal melanoma
WT: wild‐type

## 1 Introduction

RAS, as an early step in the phosphatidylinositol 3‐kinase (PI3K)–AKT signaling pathway, plays a fundamental role in oncogenesis [1, 2, 3]. Proteins in RAS‐related super‐families, particularly RAS GTPase‐activating proteins (RasGAPs), may be similarly important in oncogenesis [4, 5, 6] as exemplified by NF1 [7]. RAS protein activator‐like 1 (RASAL1) is such a RasGAP that converts active GTP‐bound RAS to inactive GDP‐bound form, attenuating the initiation of the PI3K pathway signaling [8, 9, 10]. PTEN counteracts the PI3K pathway signaling by acting as a phosphatase to dephosphorylate phosphoinositides [11]. It thus seems that RASAL1 and PTEN may act cooperatively to constrain the PI3K pathway signaling from overactivation, forming uniquely a dual oncogenesis‐suppressing mechanism.

Underexpression of *RASAL1* was associated with colorectal and gastric cancer progression [12, 13] while its overexpression suppressed the growth of gastric cancer cells [14] and thyroid cancer cells [15]. *RASAL1* hypermethylation was associated with activation of the RAS signaling in thyroid cancer [8], hepatocellular cancer, and gastric cancer [16, 17]. Some deleterious mutations in *RASAL1* were reported in follicular thyroid cancer (FTC) and anaplastic thyroid cancer (ATC) with *in vitro* confirmation of their oncogenic consequences, identifying *RASAL1* for the first time as a tumor suppressor gene in a human cancer (thyroid cancer) [8, 18]. Yet, broad genetic, clinical, and definitive functional evidence establishing *RASAL1* as a prominent general human tumor suppressor gene is lacking. Here, we used human cancer genetic and clinical data to explore the broad tumor suppressor gene candidacy of *RASAL1* and its clinical significance and used genetic mouse models to functionally confirm and establish its tumor suppressor function—particularly with respect to the *PTEN* status.

## 2 Materials and methods

### 2.1 Molecular and clinical data

Data on whole‐exome mutation, gene copy number, RNA sequencing, reverse phase protein array (RPPA), and clinical outcomes were all obtained from TCGA Pan‐Cancer Atlas (https://gdc.cancer.gov/about-data/publications/pancanatlas). Level‐4 GISTIC 2.0 results were used as inputs to analyze gene copy alterations. Mutations annotated with “Silent,” “RNA,” “Intron,” “3′UTR,” “5′UTR,” “3′Flank,” and “5′Flank” were excluded. Discrete copy number calls provided by GISTIC 2.0 were used to determine copy number alterations: −2, nullizygous deletion; −1, hemizygous deletion; 0, neutral; 1, gain; 2, high‐level amplification. Both nullizygous and hemizygous losses were treated as gene copy number loss.

### 2.2 Cancer gene database

The list of genes analyzed was from the cancer gene census (CGC) database (http://cancer.sanger.ac.uk/cancergenome/projects/census/), containing 723 oncogenes and tumor suppressor genes.

### 2.3 Statistical analysis

Fisher's exact test was used to evaluate the mutually exclusive and co‐occurred associations between *RASAL1* and other known cancer genes. *P* values were adjusted by the Bonferroni–Hochberg procedure with the false discovery control. For protein analysis (e.g., pAKT), *Z*‐score normalization was performed for each cancer type before pooling the data from different cancer types. Two‐sided Student's *t*‐test was used to compare normalized protein levels. Chi‐squared or Fisher's exact tests were used as appropriate to examine the associations between molecular alterations and clinical outcomes of cancers. Survival probability was estimated by Kaplan–Meier analysis and log‐rank test to compare the differences between Kaplan–Meier survival curves of patients with various genetic alterations. All statistical analysis was performed by R program and visualized with rstudio (PBC, Boston, MA, USA) and prism8 (Graphpad Software, Boston, MA, USA). The gene co‐occurred network was generated by the cytoscape (National Human Genome Research Institute, Bethesda, MD, USA) software.

### 2.4 Genetic knockout mouse study

A global *Rasal1* knockout (KO) mouse colony was generated using CRISPR‐CAS technology (Applied StemCell, Inc., Milpitas, CA, USA). Deletion of a 47‐bp fragment in exon 2 of *Rasal1* was achieved to cause downstream frame shift, producing a premature STOP codon. The sequences of the positions of gRNAs were mRasal1.g6 (5′‐CTGTGCGACATGGCCAAGAGCGG‐3′) and mRasal1.g11 (5′‐GGGACGAGCACTGCCCGCCAAGG‐3′). To confirm *Rasal1‐*KO, we used PCR with forward primer 5′‐CTACCTGTAGCTCTGGACGCATG‐3′ and reverse primer 5′‐CGCACCTGCTAAGCCTGG‐3′ to amplify a 398‐bp fragment expected in the wild‐type (WT) allele, a 351‐bp fragment in the KO allele, confirmed by Sanger sequencing of the PCR products. The *Rasal1* KO was also confirmed by Western blotting, showing complete loss of the ~ 90‐kD Rasal1 protein band in nullizygous KO mice. Western blotting analyses were performed using proteins derived from thyroid tissues through the Western blotting procedures described previously [19]. Primary antibodies, including anti‐RASAL1 (B‐2), anti‐phospho‐Akt1/2/3 (Ser 473), anti‐phospho‐Akt (Thr 308), and anti‐β‐actin (C‐4), were purchased from Santa Cruz Biotechnology (Santa Cruz, CA, USA). The anti‐phospho‐ERK1/2 (Thr202/Tyr204) antibody was purchased from Cell Signaling Technology (Beverly, MA, USA). HRP‐linked secondary antibodies, including anti‐mouse IgG (7076S) and anti‐rabbit IgG (7074S), were purchased from Cell Signaling Technology.

We purchased hemizygous *Pten* KO mice (42059‐JAX|mPTEN^3‐5^) from MMRRC (Mutant Mouse Resource & Research Centers) for crossing with *Rasal1* KO mice.

One hundred and fifty mice (71 male and 79 female) with a median age of 6.5 months were included in the analysis. All animals were housed and cared in an SPF‐grade animal facility, under the supervision of the Johns Hopkins Animal Care and Use Center (Protocol # MO15M31). This study was approved by the Institutional Review Board of the Johns Hopkins University School of Medicine.

## 3 Results

### 3.1 *RASAL1* is widely altered genetically in human cancers

**Fig. 1:**
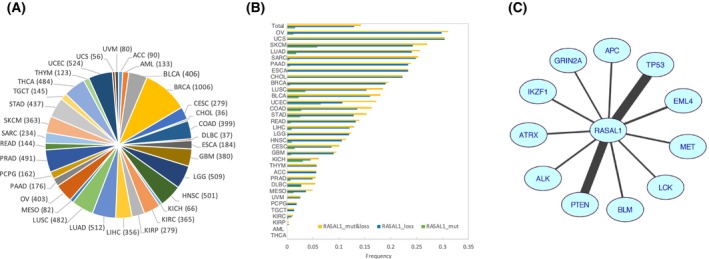
Summary of the cancer types and frequencies of the genetic alterations of *RASAL1* in 33 types of cancer. (A) Cancer types studied from TCGA database and their full names and corresponding abbreviations. The value in the parentheses represents the number of cases. (B) Presentation of the frequencies of genetic alterations of *RASAL1*. For each cancer type, yellow bar represents the collective frequency of mutation and copy number loss of *RASAL1*, the blue bar represents the frequency of copy number loss, and the green bar represents the frequency of mutations. The first group, labeled as “Total,” shows the overall frequencies of genetic alterations of *RASAL1* among the 33 types of cancer of the entire cohort of the patients. (C) Genes whose mutations showed concurrence with *RASAL1* alterations. Shown are 11 genes whose mutations frequencies were > 5% and concurred with *RASAL1* alterations in all cancer samples. The width of the line linking each gene pair is calculated by the −log (*P*) (*P* is the adjusted *P* value calculated for the strength of concurrence between the pair of genes by fisher exact test). The more significant the adjusted *P* value is, the wider the line linking the pair of genes is. The abbreviations of the cancer names are as defined in Panel A and Table S1.

We analyzed 9924 cases of cancer from 33 cancer types in TCGA (Table S1; Fig. 1A). Eight cancer types with copy number loss at a frequency > 20% showed a significant correlation between mRNA level and copy number of *RASAL1* (Fig. S1), demonstrating the functional significance of *RASAL1* copy loss. Copy loss and mutations of *RASAL1* were mutually exclusive (*P* = 0.0019, Fisher's exact test), being 12.91% and 1.52% (Table S1), respectively, suggesting their equal deleterious nature expected for a tumor suppressor gene. We thus treated mutation and copy loss collectively as deleterious genetic alterations for *RASAL1*. Their collective frequencies were > 10% in 19 cancer types, > 15% in 14 cancer types, > 20% in eight cancer types, >25% in five cancer types, and > 30% in two cancer types, with an average of 14.25% (1414/9924) in the 33 cancer types (Fig. 1B and Table S1).

### 3.2 Alterations of *RASAL1* commonly concur with those of *PTEN* or *TP53*

Analysis in the 9924 cancers for 724 known cancer genes in the Cancer Gene Census (CGC) database [20] revealed that mutations in 11 genes, whose mutation frequencies were > 5%, concurred with *RASAL1* alterations (adjusted *P* < 1.00E‐05, Fisher's exact test, Fig. 1C). The association between alterations of *RASAL1* and those of *PTEN* or *TP53* was most robust (Fig. 1C, Table S2). Specifically, 52.97% of the cancers with *RASAL1* alterations versus 33.07% of the cancers without *RASAL1* alterations had *PTEN* alterations (*P* = 4.02E‐47, Table S2). *PTEN* copy loss and mutations were both shown to activate the PI3K pathway [2, 21] and were collectively treated here as deleterious genetic alterations. Similarly, 64.78% of cancers with *RASAL1* alterations versus 33.62% of cancers without *RASAL1* alterations had *TP53* mutations (*P* = 2.43E‐110, Table S2). Thus, like *PTEN* and *TP53* alterations, *RASAL1* alterations had a wide distribution and, moreover, concurred with the former broadly in cancers (Table S3). Genetic alterations of *PTEN* and *TP53* also concurred (*P* = 2.86E‐46), consistent with previous findings that *TP53* interacted with the PI3K pathway signaling [22, 23].

### 3.3 *RASAL1* alterations activate the PI3K pathway

**Fig. 2:**
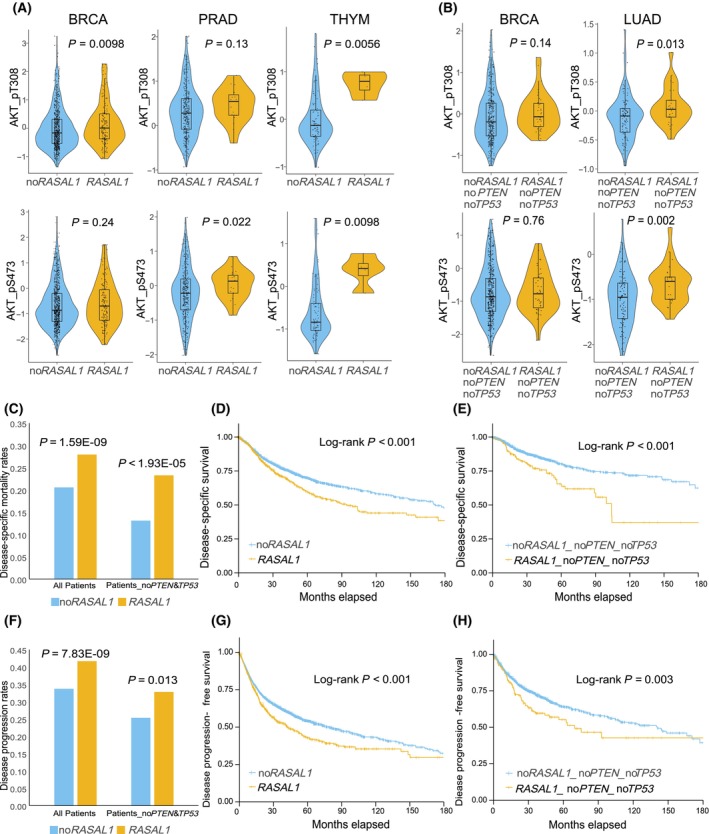
Association between *RASAL1* alterations and the PI3K/AKT pathway activation and poor clinical outcomes of cancer. (A) Association between *RASAL1* alterations and AKT phosphorylation (two‐sided Student's *t*‐test). Shown are the results in breast cancer, prostate adenocarcinoma and thymoma. The thick black bar in the middle represents the quartile range and the thin black line extending from it represents the 95% confidence interval. (B) Association between *RASAL1* alterations and AKT phosphorylation in patients without *PTEN* and *TP53* mutations in breast cancer and lung adenocarcinoma (two‐sided Student's *t*‐test). (C) Association between *RASAL1* alterations and disease‐specific mortality on the analyses of all patients or patients without *PTEN* and *TP53* alterations (chi‐squared test). (D) Effects of *RASAL1* alterations on Kaplan–Meier disease‐specific survival curves on the analysis of all patients. (E) Effects of *RASAL1* alterations on Kaplan–Meier disease‐specific survival curves on the analysis of patients without PTEN and TP53 alterations. (F) Effects of *RASAL1* alterations on disease progression rates on the analyses of all patients or patients without *PTEN* and *TP53* alterations (chi‐squared test). (G) Effects of *RASAL1* alterations on Kaplan–Meier disease progression‐free survival curves on the analysis of all patients. (H) Effects of *RASAL1* alterations on Kaplan–Meier disease progression‐free survival curves on the analysis of patients without *PTEN* and *TP53* alterations. Definitions of genotypes: no*RASAL1*, no *RASAL1* alterations; *RASAL1*, there were *RASAL1* alterations; no*PTEN*, no *PTEN* alterations; no*TP53*, no *TP53* alterations; no*PTEN*&*TP53*, no *PTEN* and *TP53* alterations; no*RASAL1*_no*PTEN*_no*TP53*, no alterations in *RASAL1*, *PTEN* and *TP53*; *RASAL1*_no*PTEN*_no*TP53*, there were *RASAL1* alterations, but no *PTEN* and *TP5*3 alterations. The name abbreviations for various cancer types are as defined in Fig. 1 and Table S1.

Analysis of the reverse phase protein array (RPPA) data revealed that cancers with *RASAL1* alterations showed higher levels of AKT phosphorylation in the PI3K pathway (pAKT, pS473 and pT308) than cancers without *RASAL1* alterations in several cancers (Fig. 2A), such as breast cancer (BRCA) (*P*_pT308 = 0.0098, *P*_pS473 = 0.24, two‐sided Student's *t*‐test), prostate adenocarcinoma (*P*_pT308 = 0.13, *P*_pS473 = 0.022), and thymoma (*P*_pT308 = 0.0056, *P*_pS473 = 0.0098). The association between AKT phosphorylation status and *RASAL1* alterations for all cancers is shown in Figs S2 and S3. In cases without *PTEN* and *TP5*3 alterations, the association between *RASAL1* alterations and pAKT still showed significance or a trend in some cancer types, such as BRCA (*P*_pT308 = 0.14, *P*_pS473 = 0.76) and lung adenocarcinoma (*P*_pT308 = 0.013, *P*_pS473 = 0.002) (Fig. 2B). Thus, *RASAL1* alterations had serious functional consequences to the PI3K pathway signaling. ERK phosphorylation (MAPK‐pT202‐Y204) in the MAPK pathway was not significantly affected in any cancer type (Fig. S4), and the ERK2 protein level was affected only in OV, and also affected in BRCA and UCEC in the absence of RAS gene mutations (Figs S5 and S6).

### 3.4 *RASAL1* alterations are associated with poor clinical outcomes of cancer

The overall disease‐specific mortality rate was 21.63% (2031/9389) while mortality was 27.93% (377/1350) with *RASAL1* alterations versus 20.57% (1654/8039) without *RASAL1* alterations (*P* = 1.59E‐09, chi‐squared test, Fig. 2C). In patients without *PTEN* and *TP53* alterations, the difference in mortality was relatively even more striking, being 23.28% (54/232) with *RASAL1* alterations versus 13.11% (498/3798) without *RASAL1* alterations, although the absolute mortality rates were lower when excluding *PTEN* and *TP53* alterations (*P* = 1.93E‐05, Fig. 2C). Kaplan–Meier analyses showed an accelerated decline in disease‐specific survival with *RASAL1* alterations compared with that without *RASAL1* alterations (HR = 1.33, Log‐rank *P* < 0.001, Fig. 2D); this effect of *RASAL1* alterations was relatively even more pronounced in patients without *PTEN* and *TP53* alterations (HR = 1.88, Log‐rank *P* < 0.001, Fig. 2E).

The overall disease progression rate was 41.65% (584/1402) with *RASAL1* alterations versus 33.67% (2800/8315) without *RASAL1* alterations (*P* = 7.83E‐09, chi‐squared test, Fig. 2F). When excluding *PTEN* and *TP53* alterations, disease progression rate was 32.77% (78/238) with *RASAL1* alterations vs 25.30% (984/3889) without *RASAL1* alterations (*P* = 0.013, Fig. 2F). Kaplan–Meier analyses of all cancers showed an accelerated decline in disease progression‐free survival with *RASAL1* alterations compared with that without *RASAL1* alterations (HR = 1.28, *P* < 0.001, Fig. 2G). Such effects of *RASAL1* alterations were also observed in patients without *PTEN* and *TP53* alterations (HR = 1.41, *P* = 0.003, Fig. 2H). An accelerated decline in disease‐specific survival or disease progression‐free survival was also observed with *RASAL1* alterations in some cancer types when individually analyzed (Fig. S7).

### 3.5 Concurrent *RASAL1* and *PTEN* alterations cooperatively activate the PI3K pathway and aggravate poor clinical outcomes

**Fig. 3:**
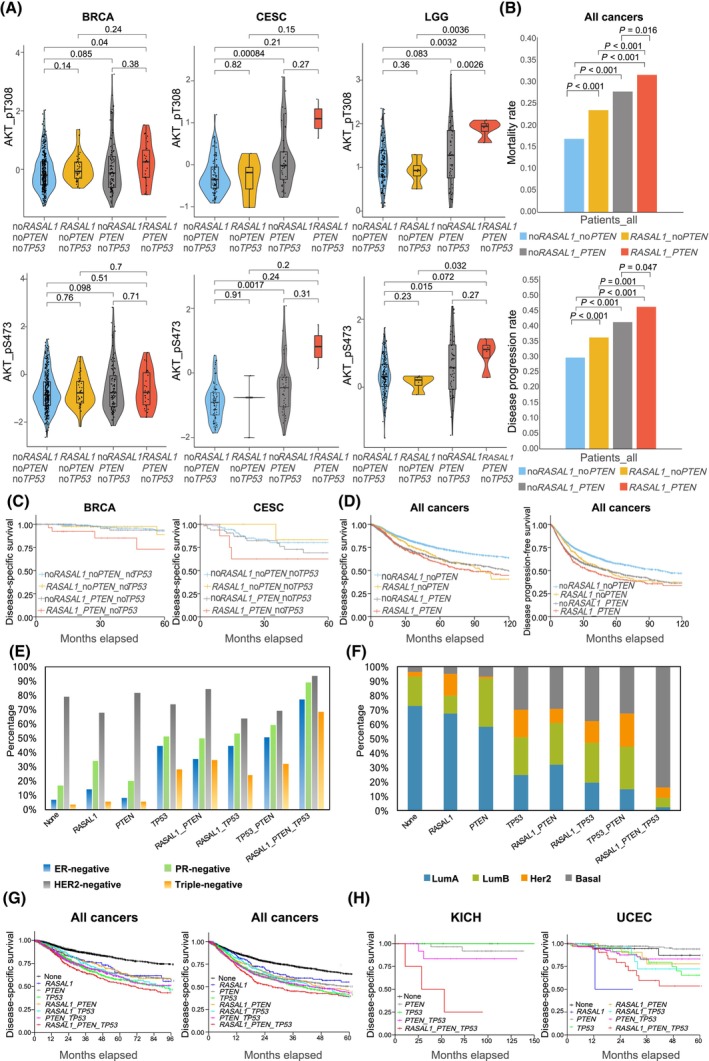
Cooperation of concurrent *RASAL1* and *PTEN* alterations in synergistically activating the PI3K pathway (AKT phosphorylation) and promoting aggressive clinical outcomes. (A) Cooperative effects of concurrent *RASAL1* and *PTEN* alterations on AKT phosphorylation in breast cancer, cervical cancer, and low‐grade glioma patients without *TP53* mutations (two‐sided Student's *t*‐test). The thick black bar in the middle represents the quartile range and the thin black line extending from it represents the 95% confidence interval. (B) Cooperative effects of concurrent *RASAL1* and *PTEN* alterations on disease‐specific mortality rates and disease progression rates on the analyses of all cancer patients (chi‐squared test). (C) Cooperative effects of concurrent *RASAL1* and *PTEN* alterations on Kaplan–Meier disease‐specific survival curves in patients with breast cancer or cervical cancer patients without *TP53* mutations. (D) Cooperative effects of concurrent *RASAL1* and *PTEN* alterations on Kaplan–Meier disease‐specific survival curves and disease progression‐free survival curves on the analyses of all cancer patients. (E) Status of ER, PR, and HER2 expression with various genotypes of *RASAL1*, *PTEN*, and *TP53* alterations in breast cancer (chi‐squared test). (F) Distribution of breast cancer subtypes among various genotypes of *RASAL1*, *PTEN* and *TP53* alterations (chi‐squared test). (G) Kaplan–Meier disease‐specific survival curves and disease progression‐free survival curves with various genotypes of *RASAL1*, *PTEN*, and *TP53* alterations on the analyses of all cancer patients. (H) Kaplan–Meier disease‐specific survival curves in various genotypes of *RASAL1*, *PTEN* and *TP53* alterations in patients with chromophobe renal cell carcinoma or uterine corpus endometrial carcinoma. The definitions of various genotypes of *RASAL1*, *PTEN*, and *TP53* alterations are as defined in Fig. 2.

Given that TP53 could interact with the PI3K pathway signaling [22, 23], we compared four genotypes without *TP53* mutations: no *RASAL1* and *PTEN* alterations (no*RASAL1*_no*PTEN_* no*TP53*), only *RASAL1‐*altered (*RASAL1*_no*PTEN_*no*TP53*), only *PTEN‐*altered (no*RASAL1*_*PTEN_*no*TP53*), and both *RASAL1‐* and *PTEN‐*altered (*RASAL1*_*PTEN_* no*TP53*). In BRCA, which had the largest sample size, the *RASAL1*_*PTEN_noTP53* group showed the highest AKT_pT308 level, significantly higher than that in the no*RASAL1*_no*PTEN_*no*TP53* group (*P* = 0.04, two‐sided Student's *t* test, Fig. 3A); the only *RASAL1*‐ or only *PTEN*‐altered group showed a higher trend compared with the no*RASAL1*_no*PTEN_*no*TP53* group (*P* = 0.14 and 0.085, Fig. 3A). The AKT‐pS473 levels showed similar patterns (Fig. 3A). In cervical cancer (CESC), the *RASAL1*_*PTEN_*no*TP53* group showed the highest AKT_pT308 and AKT_pS473 levels compared with other groups albeit insignificant with limited number of cases. In low grade glioma (LGG), the *RASAL1*_*PTEN_*no*TP53* group showed the highest AKT_pT308 level, higher than any of the other three groups (*P* = 0.0032, 0.0036, and 0.0026, Fig. 3A), with no difference between the only *RASAL1‐* or only *PTEN‐*altered group and the no*RASAL1*_no*PTEN_*no*TP53* group (*P* = 0.36 and 0.083).

Similarly, on the analyses of all cancers, the *RASAL1*_*PTEN* group showed the highest disease‐specific mortality rate, which was higher than that of the no*RASAL1*_no*PTEN*, *RASAL1*_no*PTEN*, or no*RASAL1*_*PTEN* group (*P* = 1.31E‐19, 1.87E‐04 and 0.016), with similar genetic‐dependent patterns of disease progression rates (*P* = 1.80E‐21, 0.001, and 0.047) (Fig. 3B). Overall, disease progression and mortality rates were 46.36% and 31.72% with the genetic duet of *RASAL1* and *PTEN* alterations versus 29.80% and 16.93% with neither mutation (HR 1.64, 95% CI 1.46–1.84 and 1.77, 95% CI 1.53–2.05), respectively. On Kaplan–Meier analyses of BRCA, the *RASAL1*_*PTEN_*no*TP53* group showed the most accelerated decline in disease‐specific survival compared with the no*RASAL1*_no*PTEN_*no*TP53*, *RASAL1*_no*PTEN_*no*TP53* or no*RASAL1*_*PTEN_*no*TP53* group (HR = 3.46, 1.68 and 2.28; *P* = 0.015, 0.46 and 0.17, Fig. 3C). Analyses of CESC with limited sample size of the *RASAL1*_*PTEN_*no*TP53* group showed similar trends (HR = 2.08, 3.81 and 1.52; *P* = 0.23, 0.21 and 0.5, Fig. 3C).

On the analyses of all cancers, the *RASAL1*_*PTEN* group showed the most accelerated decline and the *RASAL1*_no*PTEN* or no*RASAL1*_*PTEN* showed an intermediate decline in disease‐specific survival (DSS) and disease progression‐free survival (PFS), when compared with the no*RASAL1*_no*PTEN* group (HR = 1.769, 1.660 and 1.445 for DSS; HR = 1.638, 1.476 and 1.307 for PFS, all *P* values < 1.00E‐04, Fig. 3D). The single genetic alteration groups *RASAL1*_no*PTEN* and no*RASAL1*_*PTEN* showed no difference in DSS and PFS (*P* values = 0.1 and 0.07 for DSS and PFS, Fig. 3D). Thus, concurrent *RASAL1* and *PTEN* alterations cooperatively aggravated cancer aggressiveness.

### 3.6 Concurrence of triple gene alterations of *RASAL1*, *PTEN*, and *TP53* is associated with poorest clinical outcomes

We investigated this using BRCA as it had the largest sample size and high frequency of triple genetic alterations. As shown in Fig. 3E, the *RASAL1‐*altered group showed higher estrogen receptor (ER)‐negative and progesterone receptor (PR)‐negative rates compared with the group without *RASAL1* alterations (*P* = 0.095 and 6.27E‐03, chi‐squared test). The *RASAL1*_*PTEN* group showed higher ER‐negative and PR‐negative rates compared with the only *PTEN‐*altered group (*P* = 1.90E‐04 and 8.56E‐04) or only *RASAL1‐*altered group (*P* = 0.043 and 0.215). ER‐ and PR‐negative rates were 77.4% and 88.7%, respectively, in the *RASAL1*_*PTEN*_*TP53* triple‐altered group, strikingly higher than any of other groups, such as the only *RASAL1‐*altered or only *PTEN‐*altered group (*P* < 3.50E‐03 and < 2.00E‐04 for both). A high HER2‐negative rate of 93.75% was found in *RASAL1*_*PTEN*_*TP53* triple‐altered group. We also found a high rate of 83.7% of the basal‐like BRCA, the most aggressive type of BRCA [24], in the *RASAL1*_*PTEN*_*TP53* group vs 29.0%, 37.5%, and 32.4% in the *RASAL1_PTEN*, *RASAL1_TP53*, and *PTEN_TP53* groups, respectively (*P* < 1.00E‐03 for all) (Fig. 3F). These results are consistent with the previous report that most of the basal‐like BRCA were triple‐negative [25]. We also examined the potential cooperative effect of concurrent *RASAL1*, *PTEN*, and *TP53* alterations on the PI3K‐AKT pathway. Our findings revealed that *TP53* alteration alone did not have any effect on AKT phosphorylation. The triple genetic alteration group also did not affect significantly AKT phosphorylation when compared with *RASAL1*_*PTEN* group (Fig. S8, Tables S4 and S5). Therefore, although the triple genetic alteration group had a robustly unfavorable prognosis in breast cancer, this poor prognosis was not solely achieved through the PI3K‐AKT pathway. Instead, it is likely the result of the synergistic effects between the PI3K‐AKT pathway activated by the coexisting *RASAL1* and *PTEN* alterations and other pathway(s) promoted by the *TP53* alterations.

On the analysis of all cancers, the group without gene alterations displayed the lowest decline and the *RASAL1*_*PTEN_TP53* group displayed the sharpest decline in disease‐specific or disease progression‐free survivals (Fig. 3G). Disease progression and mortality rates were 47.65% and 34.46% with the trio of *RASAL1*, *PTEN*, and *TP53* alterations versus 25.30% and 13.11% with no mutation (HR 2.21, 95% CI 1.92–2.56 and 2.76, 95% CI 2.31–3.30), respectively. Analysis of individual cancer types with relatively large sample size showed similar results (Fig. 3H). Thus, alterations of the three genes *RASAL1*, *PTEN*, and *TP53* had superimposed adverse effect on clinical outcomes of cancer. In particular, the genetic trio sharply increased breast cancer's risk to be triple‐negative, being 68.75% versus 3.83% with no mutation (RR 17.94, 95% CI 9.60–33.51). Thus, the triple concurrence of *RASAL1*, *PTEN*, and *TP53* alterations is a robust genetic biomarker of the aggressive triple‐negative basal‐like BRCA.

### 3.7 The dual loss of *Rasal1* and *Pten* cooperatively drives oncogenecity and cancer metastasis in genetic knockout mouse models

**Fig. 4:**
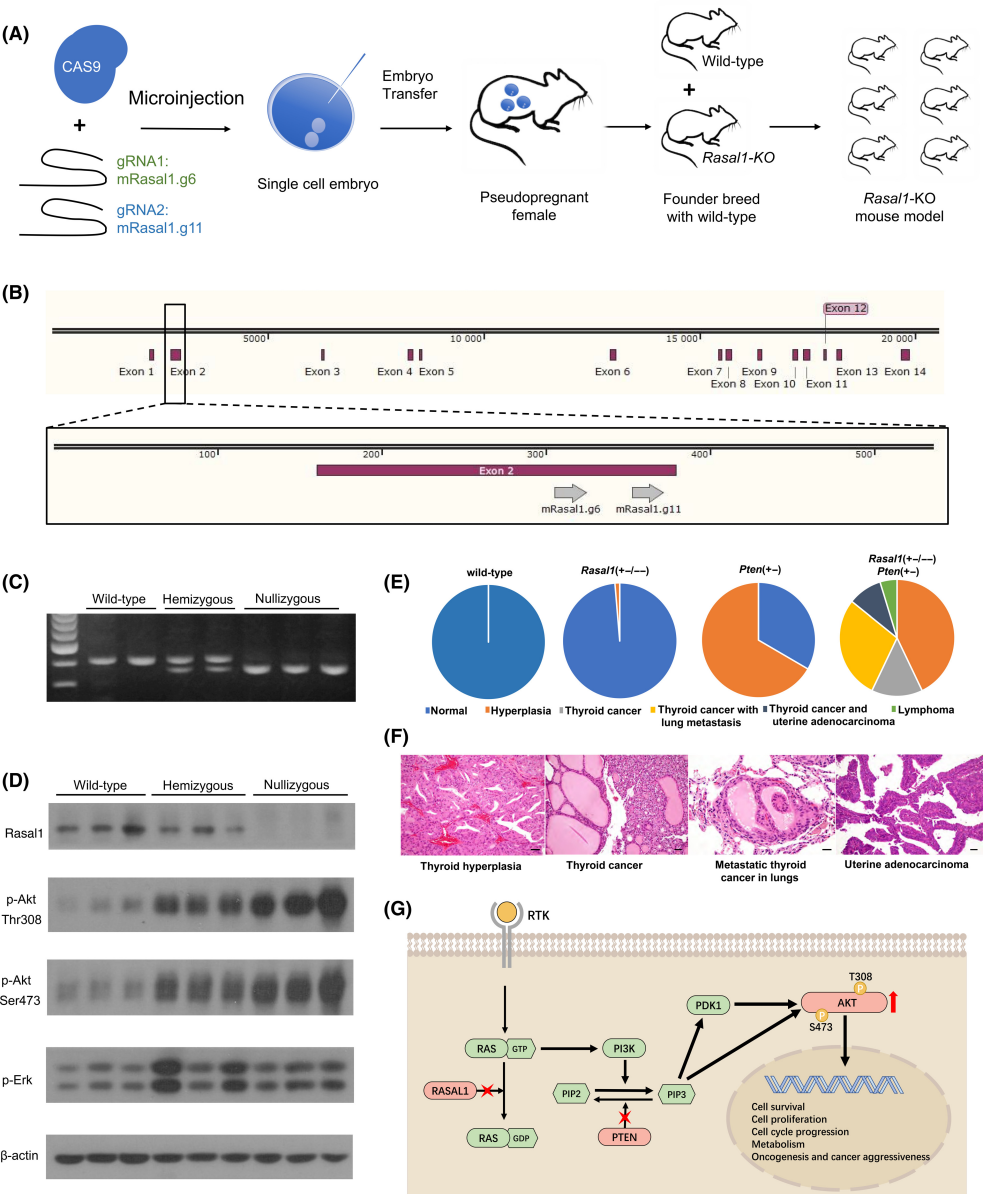
Genetic knockout mice show the tumor suppressor role of *RASAL1* and the cooperation between *RASAL1* and *PTEN* defects in driving oncogenesis and cancer aggressiveness, as found in humans. (A) Generation of the global *Rasal1*‐knockout mouse model and colony development. (B) Illustration of the location of two candidate gRNAs within Exon 2 of mRasal1 gene. (C) All mice (*n* = 150) were genotyped for the *Rasal1* gene using PCR sequencing. Representative PCR amplification patterns demonstrate a single upper band (398 bp) in wild‐type *Rasal1*+/+ mice, a single lower band (351 bp) in nullizygous *Rasal1* −/− mice, and both bands in hemizygous *Rasal1*+/− mice. (D) Western blotting analyses (*n* = 9) of the expression levels of several signaling proteins in various *Rasal1* genotypes. (E) Pie chart summary (*n* = 40, 74, 15 and 21, respectively) of the frequencies of pathological conditions in various knockout genotype mice. (F) Representative microscopic images of pathological conditions (thyroid hyperplasia (*n* = 17), thyroid cancer (*n* = 11), metastatic thyroid cancer in lungs (*n* = 6), and uterine adenocarcinoma (*n* = 2)) that developed in knockout mice. The scale bars represent 50 μm. (G) Schematic illustration of the mechanistic model in which concurrent *RASAL1* and *PTEN* alterations can cooperatively activate the PI3K pathway to promote oncogenesis and cancer aggressiveness. In this mechanism, the defect of *RASAL1* results in retention of RAS‐GTP, thus maintaining constitutive RAS activation; this initiates the signaling of the PI3K‐AKT pathway, which cannot be terminated in the presence of *PTEN* defects.

A *Rasal1* knockout (KO) mouse model was created using CRISPR‐CAS technology (Applied StemCell, Inc.). The KO procedure, the deletion position of *Rasal1*, and the deletion confirmation by PCR, Western blotting, and Sanger sequencing are shown in Fig. 4A–D and Fig. S9, respectively. The p‐Akt (Thr308) and p‐Akt (Ser473) levels were significantly higher in hemizygous KO mice and even higher in nullizygous KO mice than those in WT mice (Fig. 4D). The p‐Erk level was modestly and inconsistently increased. Thus, the Pi3k pathway was preferentially activated over the Mapk pathway in *Rasal1* KO mice.

*Rasal1*−/− mice were crossed with hemizygous *Pten* KO mice (42059‐JAX|mPTEN^3–5^) [26], producing 40 wild‐type (WT), 74 *Rasal1* +/− or −/−, 15 *Pten* +/−, and 21 *Rasal1‐Pten* double‐KO mice; they were 4–12 months old, with the median age of 6.5, 7.5, 5, and 6 months, respectively, at sacrifice. As shown in Fig. 4E, all WT mice and 98.65% (73/74) of *Rasal1* +/− or −/− mice had normal organs/tissues on whole body examination except for one *Rasal1*−/− mouse that developed focal thyroid hyperplasia. Hyperplasia was found in 66.67% (10/15) of *Pten* +/− mice, including nine in thyroid gland and one in uterus. Among the 21 *Rasal1*‐*Pten* double‐KO mice, 42.9% (9/21) developed hyperplasia (6 in thyroid gland and 3 in both thyroid gland and uterus) and 57.1% (12/21) developed cancers, including 11 thyroid cancer and 1 lymphoma. Among the mice with thyroid cancer, six had lung metastases and two had coexisting uterus adenocarcinoma. Representative microscopic images of various pathological conditions are presented in Fig. 4F. Thus, while single alteration of *Rasal1* or *Pten* had limited oncogenic effects, dual alterations of the two genes robustly promoted oncogenesis, malignant transformation, and cancer metastasis.

## 4 Discussion

We provide here broad genetic, clinical, and functional evidence firmly establishing *RASAL1* as a prominent general human tumor suppressor gene, whose alterations play a robust role in cancer aggressiveness through overactivating the PI3K pathway, particularly when uniquely concurrent with *PTEN* alterations. Given that RASAL1 and PTEN normally cooperatively constrain the PI3K pathway signaling, at an upper and lower step, respectively, it is plausible to see this robust cooperation of *RASAL1* and *PTEN* alterations in activating the PI3K pathway and promoting cancer aggressiveness. *RASAL1* alterations cause constitutive activation of RAS, initiating the PI3K pathway signaling, which will be unconstrained in the presence of defective *PTEN*, thus constantly activating the PI3K pathway signaling and driving the aggressiveness and poor clinical outcomes of human cancer (Fig. 4G). *RASAL1* has abnormalities also at epigenetic and post‐translational levels [8, 16, 17]. Among various RasGAPs, only RASAL1 expression was decreased in cancer [12]. These all further support *RASAL1* being a prominent tumor suppressor gene.

The present study provides important clinical implications on the prognostic value of genetic alterations of *RASAL1*, which were significantly associated with poor clinical outcomes, including decreased disease recurrence‐free survival and disease‐specific survival. This is even more clear with the case of the genetic duet of *RASAL1* and *PTEN* alterations, which was robustly associated with disease recurrence and disease‐specific mortality in many cancers in the present study. Previous studies reported mutual upregulation between TP53 and PTEN [22, 27] and concurrent *PTEN* and *TP53* mutations associated with cancer aggressiveness [28, 29]. Interestingly, the present study demonstrated triple concurrence of *RASAL1*, *PTEN*, and *TP53* alterations, which was associated with the poorest clinical outcomes of cancer and represented a particularly robust biomarker of BRCA being the aggressive triple‐negative basal‐like type. We have attempted to breed triple‐mutant mouse with *p53*mut/*Rasal1*‐KO/*PTEN* KO, but failed to obtain live offsprings.

Consistent with the present study, we previously found hardly mutations in *RASAL1* in papillary thyroid cancer (PTC), but found inactivating *RASAL1* mutations in FTC and ATC although *RASAL1* hypermethylation was common in PTC [8]. Cowden Syndrome, caused by *PTEN* mutations, presents characteristically with FTC and BRCA [30]. Some germline alterations in *RASAL1* were found in Cowden Syndrome patients presenting with FTC [9, 18] and germline variants in *RASAL1* were suggested to confer susceptibility to BRCA [31]. These data are consistent with our present study suggesting that deleterious alterations of *RASAL1* and *PTEN* act oncogenically alone the same line through activating the PI3k pathway.

FTC and ATC are driven by the PI3K pathway, whereas PTC is driven mainly by the MAP kinase pathway [2, 32]. *RAS* mutations preferentially activate the PI3K pathway over the MAP kinase pathway [2, 33] and occur more commonly in FTC and ATC than in PTC [2, 32]. These are consistent with the present findings that *RASAL1* alterations preferentially activated the PI3K pathway over the MAP kinase pathway and were uncommon in PTC.

## 5 Conclusion

This study provides strong and broad genetic, clinical, and functional evidence establishing *RASAL1* as another prominent general tumor suppressor gene, after *PTEN*, in the PI3K pathway. The genetic duet of concurrent *RASAL1* and *PTEN* alterations forms a unique genetic mechanism that robustly promotes oncogenesis and cancer aggressiveness through cooperatively activating the PI3K pathway, which can be further aggravated by coexisting *TP53* alterations. This unique genetic mechanism adds a new dimension to understanding how the PI3K pathway plays its fundamental role in oncogenesis, which also represents a new prognostic genetic background that may potentially be integrated into molecular‐based clinical risk stratification schemes for precision management of human cancer, such as breast cancer.

## Conflict of interest

The authors declare no conflict of interest. The results published here are partially based upon data generated by the TCGA Research Network: http://cancergenome.nih.gov/.

## Author contributions

XS, JT, and MX contributed to designing and performing the research, analyzing the data, and writing the manuscript. RL, GZ, and LR contributed to performing the research. MX conceived and supervised the project and was responsible for its overall strategy and management.

## Supporting information


**Fig. S1.** Association between *RASAL1* copy number alterations and *RASAL1* mRNA expression.
**Fig. S2.** Relationship between AKT_pT308 phosphorylation and *RASAL1* alterations.
**Fig. S3.** Relationship between AKT_pS473 phosphorylation and *RASAL1* alterations.
**Fig. S4.** Relationship between ERK phosphorylation and *RASAL1* alterations.
**Fig. S5.** Relationship between ERK2 protein expression and *RASAL1* alterations.
**Fig. S6.** Relationship between *RASAL1* alterations and ERK2 protein expression in the absence of *RAS* gene mutations.
**Fig. S7.** Kaplan–Meier analyses of the effects of *RASAL1* alterations on the survivals of patients with the indicated cancers.
**Fig. S8.** Exploration of cooperative effects of concurrent *RASAL1*, *PTEN*, and *TP53* alterations on AKT phosphorylation in BRCA.
**Fig. S9.** Successful deletion of *Rasal1* in *Rasal1* KO mice.
**Table S1.** Frequency of the genetic alterations of *RASAL1* in 33 cancer types.
**Table S2.** Coexistences between *RASAL1* alterations and *PTEN* alterations or *TP53* mutations.
**Table S3.** Association between genetic alterations of *RASAL1* and those of *PTEN* or *TP53* in individual cancer types.
**Table S4.** *P*‐value matrix of between‐group differences in AKT_pT308 phosphorylation based on *RASAL1*, *PTEN*, and *TP53* alterations in breast cancer.
**Table S5.** *P*‐value matrix of between‐group differences in AKT_pS473 phosphorylation based on *RASAL1*, *PTEN*, and *TP53* alterations in breast cancer.

## Data Availability

All data generated or analyzed during this study are included in this published article and its Supporting information files.
