# Supplementary material for: The genetic duet of concurrent RASAL1 and PTEN alterations promotes cancer aggressiveness by cooperatively activating the PI3K–AKT pathway
Source: Mol Oncol. 2024 Jul 20;19(1):248–59. doi: 10.1002/1878-0261.13701 (PMC11705815; doi:10.1002/1878-0261.13701)
Supplement: Supplementary file 1 — Fig. S1. Association between RASAL1 copy number alterations and RASAL1 mRNA expression. Fig. S2. Relationship between AKT_pT308 phosphorylation and RASAL1 alterations. Fig. S3. Relationship between AKT_pS473 phosphorylation and RASAL1 alterations. Fig. S4. Relationship between ERK phosphorylation and RASAL1 alterations. Fig. S5. Relationship between ERK2 protein expression and RASAL1 alterations. Fig. S6. Relationship between RASAL1 alterations and ERK2 protein expression in the absence of RAS gene mutations. Fig. S7. Kaplan–Meier analyses of the effects of RASAL1 alterations on the survivals of patients with the indicated cancers. Fig. S8. Exploration of cooperative effects of concurrent RASAL1, PTEN, and TP53 alterations on AKT phosphorylation in BRCA. Fig. S9. Successful deletion of Rasal1 in Rasal1 KO mice. Table S1. Frequency of the genetic alterations of RASAL1 in 33 cancer types. Table S2. Coexistences between RASAL1 alterations and PTEN alterations or TP53 mutations. Table S3. Association between genetic alterations of RASAL1 and those of PTEN or TP53 in individual cancer types. Table S4. P‐value matrix of between‐group differences in AKT_pT308 phosphorylation based on RASAL1, PTEN, and TP53 alterations in breast cancer. Table S5. P‐value matrix of between‐group differences in AKT_pS473 phosphorylation based on RASAL1, PTEN, and TP53 alterations in breast cancer. [file MOL2-19-248-s001.docx]

**Supplementary Appendix**

**Table of contents**

| Contents | Page |
| --- | --- |
| Figure S1: Association between *RASAL1* copy number alterations and *RASAL1* mRNA expression. | 2 |
| Figure S2: The relationship between AKT_pT308 phosphorylation and *RASAL1* alterations. | 3 |
| Figure S3: The relationship between AKT_pS473 phosphorylation and *RASAL1* alterations. | 5 |
| Figure S4: The relationship between ERK phosphorylation and *RASAL1* alterations. | 7 |
| Figure S5: The relationship between ERK2 protein expression and *RASAL1* alterations. | 9 |
| Figure S6:The relationship between *RASAL1* alterations and ERK2 protein expression in the absence of *RAS* gene mutations. | 11 |
| Figure S7: Kaplan-Meier analyses of the effects of *RASAL1* alterations on the survivals of patients with the indicated cancers. | 12 |
| Figure S8: Exploration of cooperative effects of concurrent *RASAL1*, *PTEN* and *TP53* alterations on AKT phosphorylation in BRCA. | 13 |
| Figure S9: Successful deletion of *Rasal1* in *Rasal1* KO mice. | 14 |
| Table S1: Frequency of the genetic alterations of *RASAL1* in 33 cancer types | 15 |
| Table S2: Co-existences between *RASAL1* alterations and *PTEN* alterations or *TP53* mutations. | 17 |
| Table S3: Association between genetic alterations of *RASAL1* and those of *PTEN* or *TP53* in individual cancer types. | 18 |
| Table S4: P-value matrix of between-group differences in AKT_pT308 phosphorylation based on *RASAL1*,*PTEN* and *TP53* alterations in breast cancer | 19 |
| Table S5: P-value matrix of between-group differences in AKT_pS473 phosphorylation based on *RASAL1*,*PTEN* and *TP53* alterations in breast cancer | 19 |

**Figure S1**. **Association between *RASAL1* copy number alterations and *RASAL1* mRNA expression.**

A positive association between *RASAL1* copy number alterations and RNA expression was seen collectively in pooled 8 cancer types whose frequency of *RASAL1* loss were higher than 20% and individually in LUAD, OV, and SKCM as indicated. −2, nullizygous deletion; −1, hemizygous deletion; 0, neutral; 1, gain; 2, high-level amplification. The abbreviations of the cancer names are as defined in Figure 1 and Table S1.


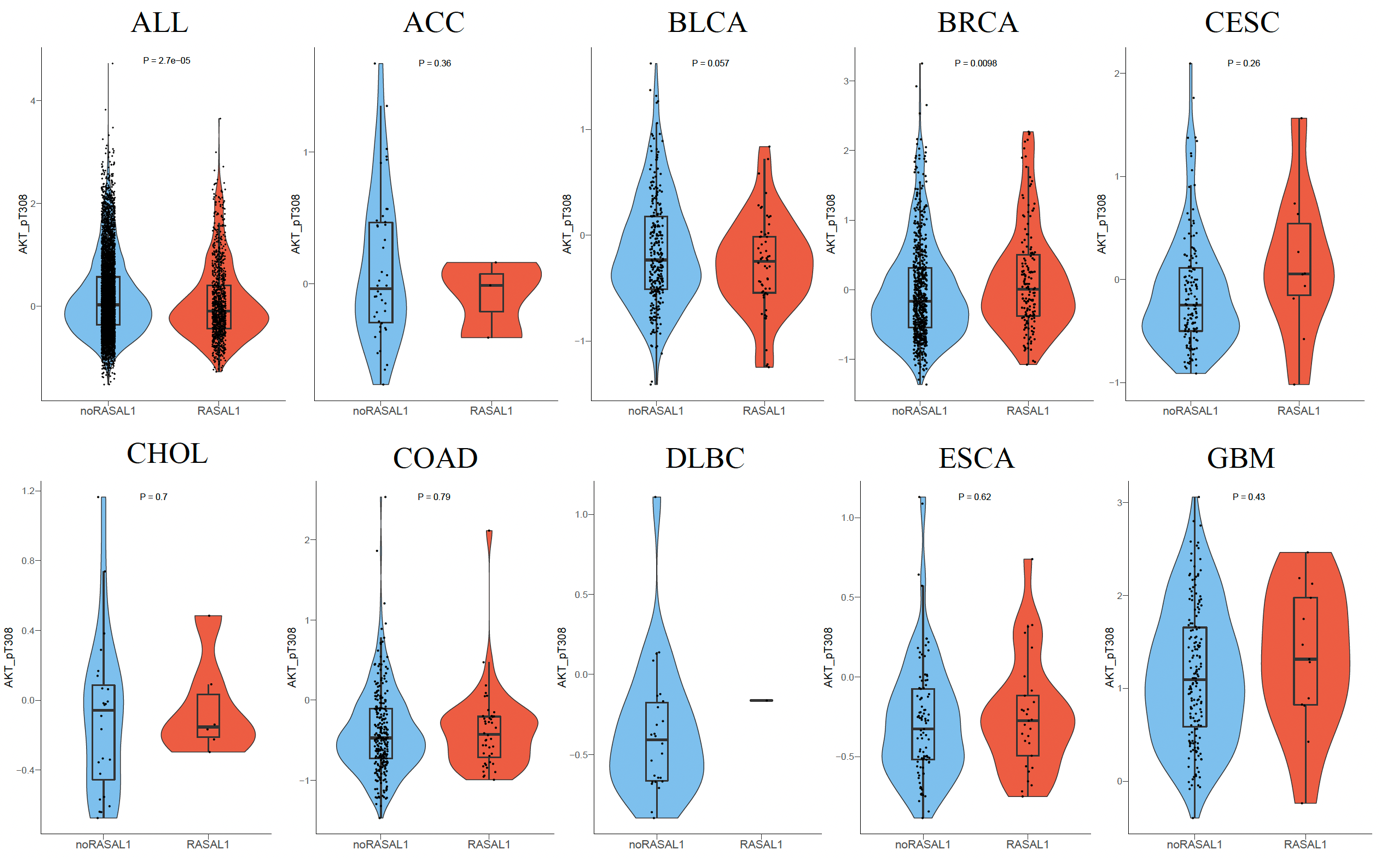


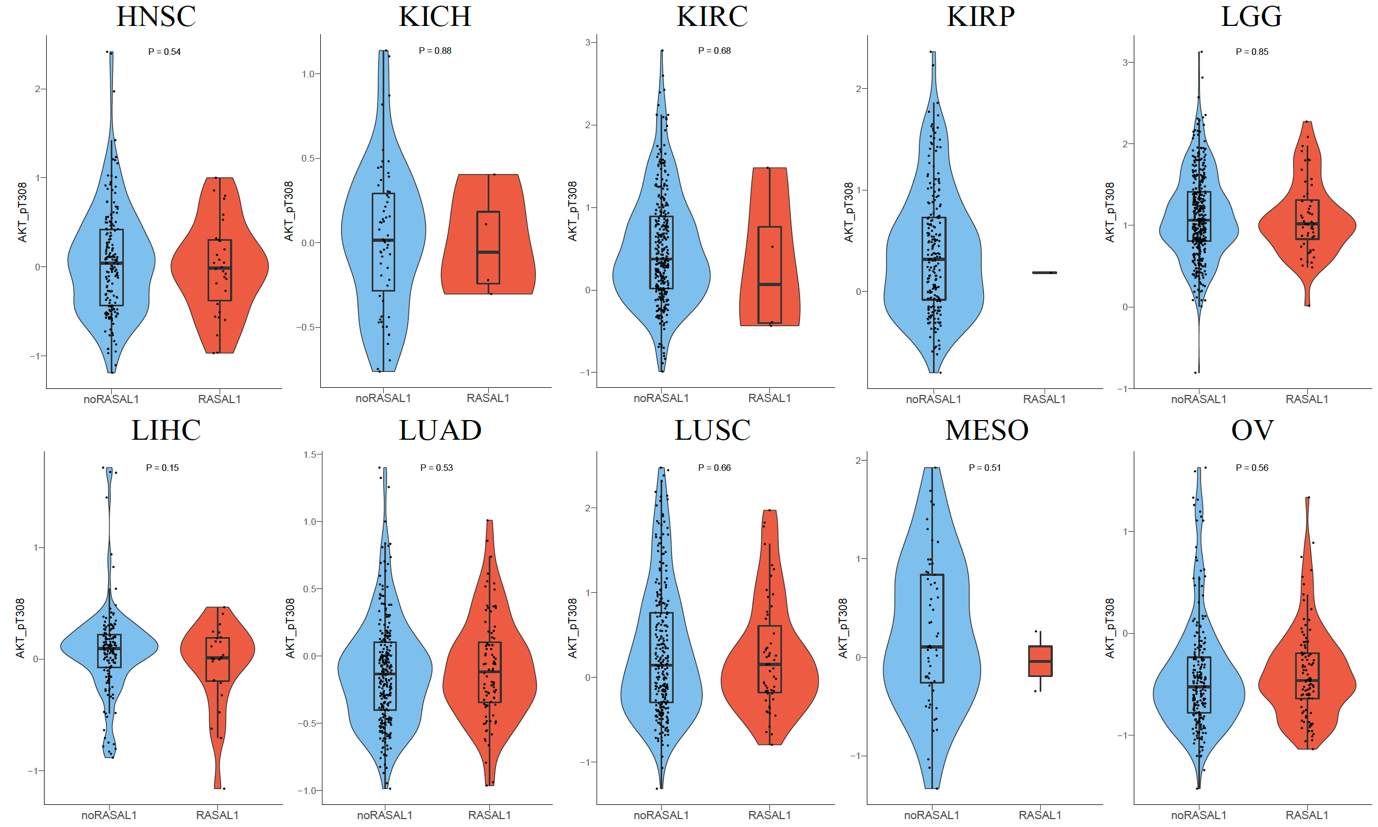


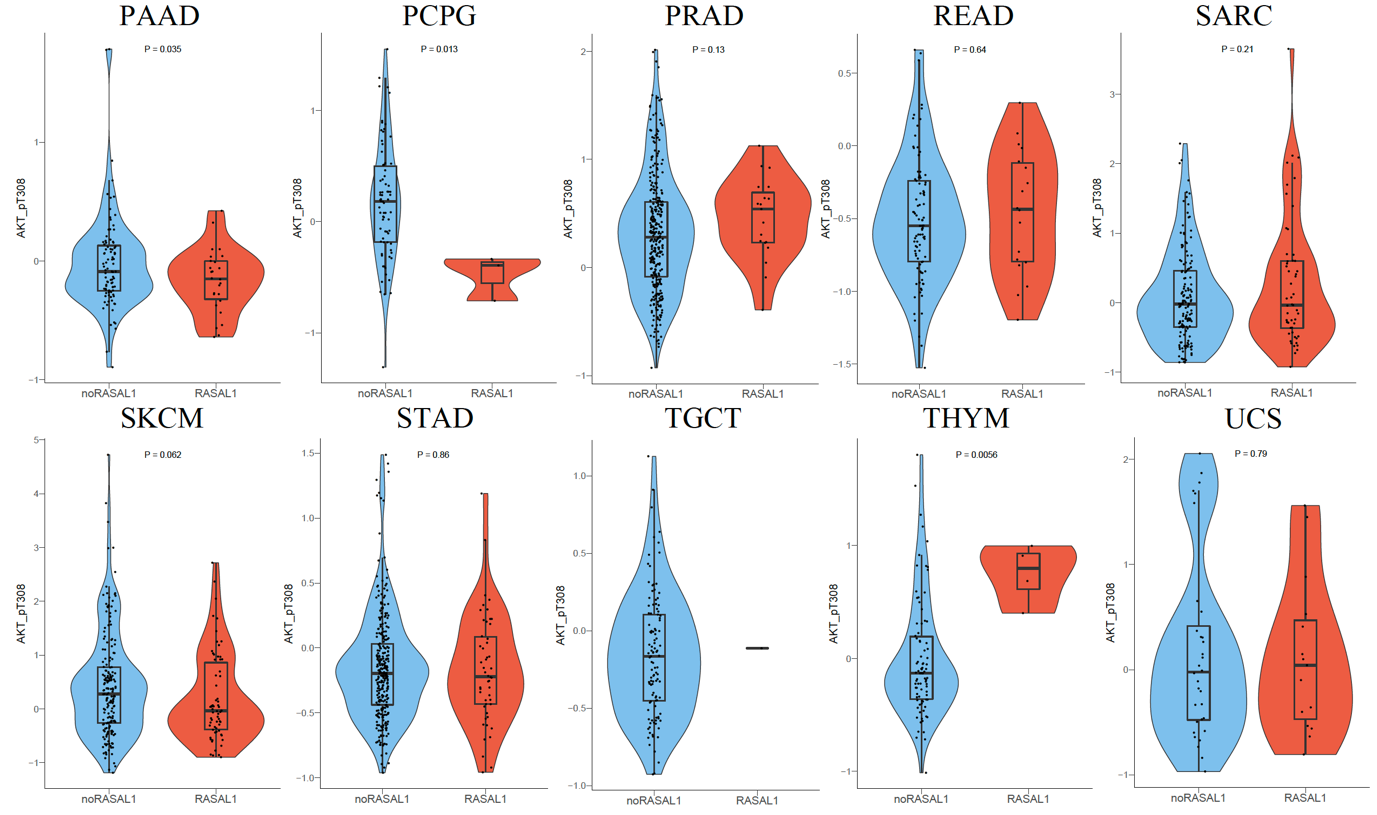


**Figure S2. The relationship between** **AKT_pT308 phosphorylation and *RASAL1* alterations**. Shown are the relationship between AKT_pT308 phosphorylation and *RASAL1* alterations in all cancers collectively and in the 30 different cancer types individually as indicated. Samples with *RASAL1* alterations showed higher levels of AKT_pT308 in the PI3K pathway than samples without *RASAL1* alterations in BRCA and THYM (P =0.0098 and 0.0056). AKT_pT308 were also found significantly downregulated in PAAD and PCPG, but the number of samples with the mutation in these two cancer types were small (P= 0.035 and 0.013). noRASAL1, cases with no *RASAL1* alterations; RASAL1, cases with *RASAL1* alterations. The abbreviations of the cancer names are as defined in Figure 1 and Table S1.


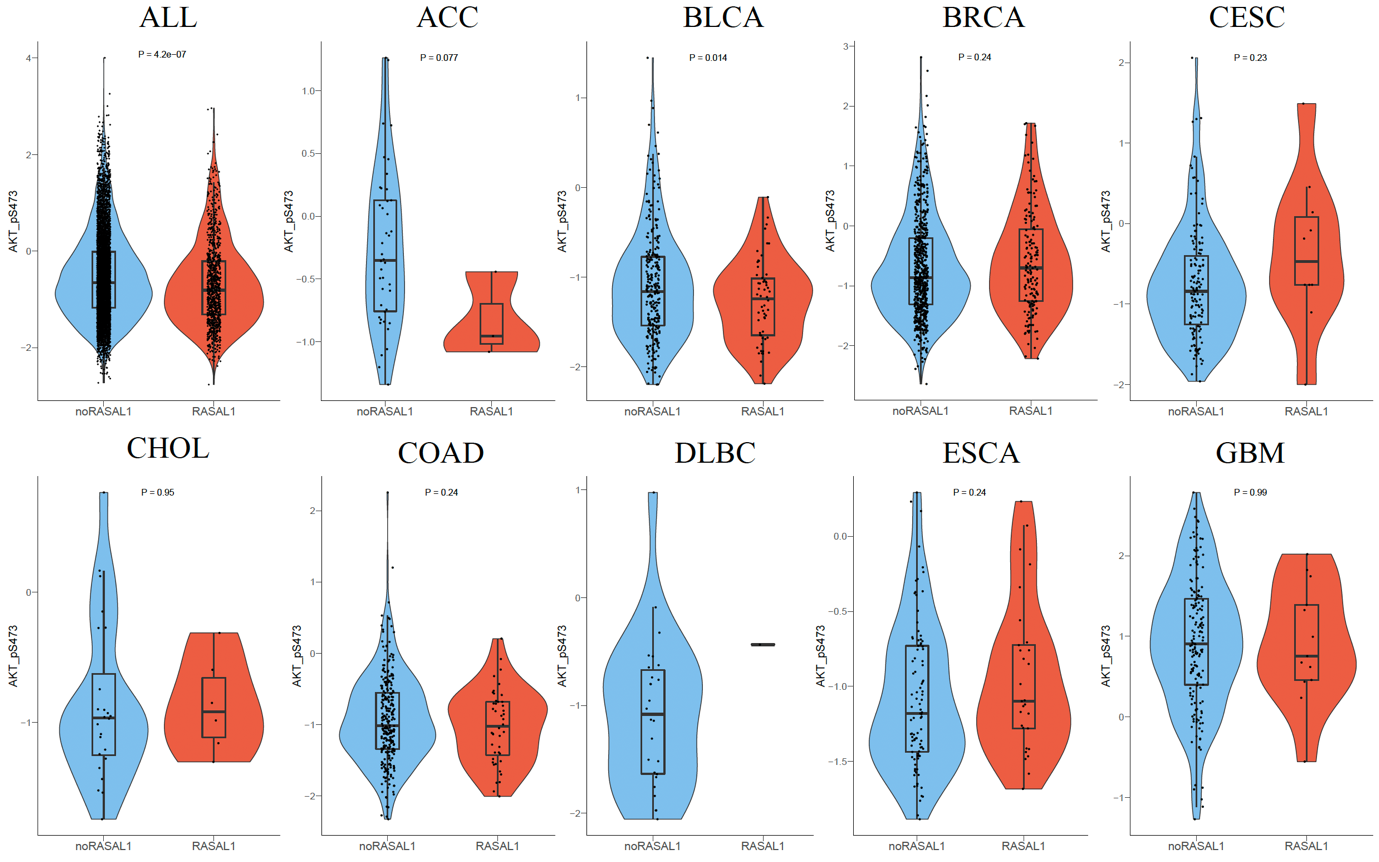


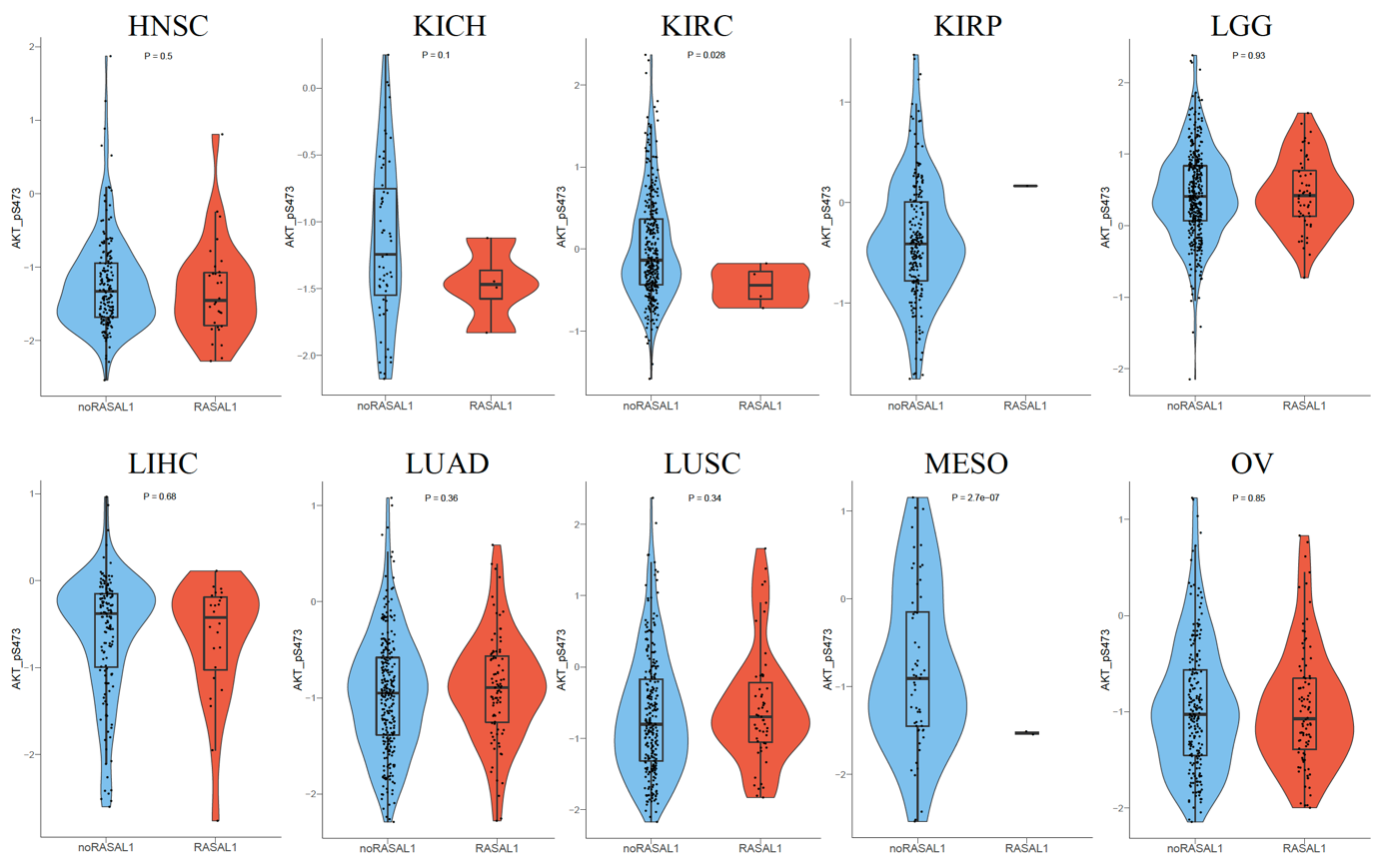


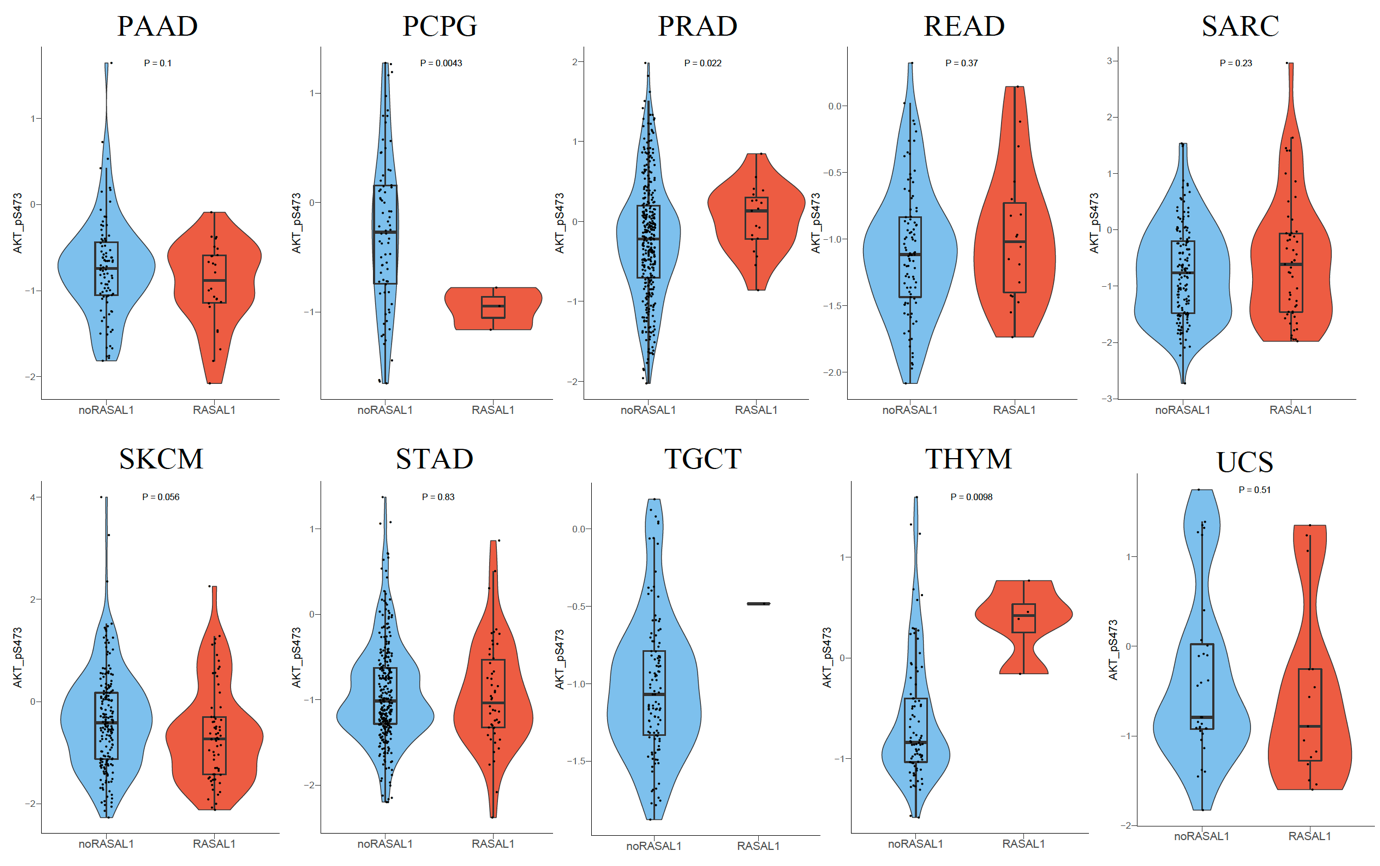

**Figure S3. The relationship between AKT_pS473 phosphorylation and *RASAL1* alterations**. Shown are the relationship between phosphorylated AKT_pS473 and *RASAL1* alterations in all cancers collectively and in the 30 different cancer types individually as indicated. noRASAL1, cases with no *RASAL1* alterations; RASAL1, cases with *RASAL1* alterations. The abbreviations of the cancer names are as defined in Figure 1 and Table S1.

ALL ACC LCA BRCA CESC

CHOL COAD DLBC ESCA GBM

HNSC KICH KIRC KIRP LGG

LIHC LUAD LUSC MESO OV

PAAD PCPG PRAD READ SARC

STAD TGCT THYM UCEC UCS

**Figure S4.** **The relationship between** **ERK phosphorylation and *RASAL1* alterations**. Shown are the relationship between phosphorylated ERK and *RASAL1* alterations in all cancers collectively and in the 30 different cancer types individually as indicated. No significant association was observed between ERK phosphorylation and *RASAL1* alterations. noRASAL1, cases with no *RASAL1* alterations; RASAL1, cases with *RASAL1* alterations. The abbreviations of the cancer names are as defined in Figure 1 and Table S1.


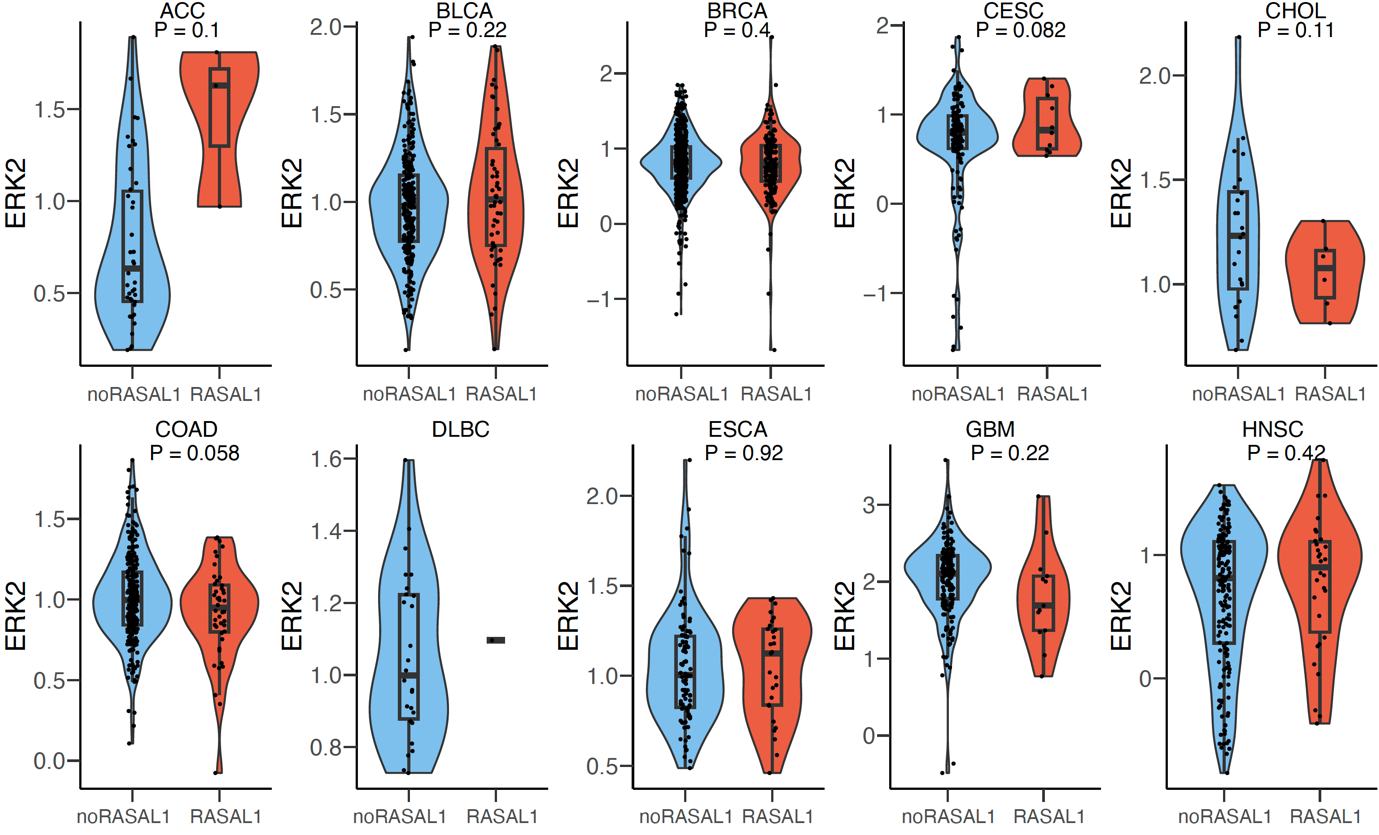


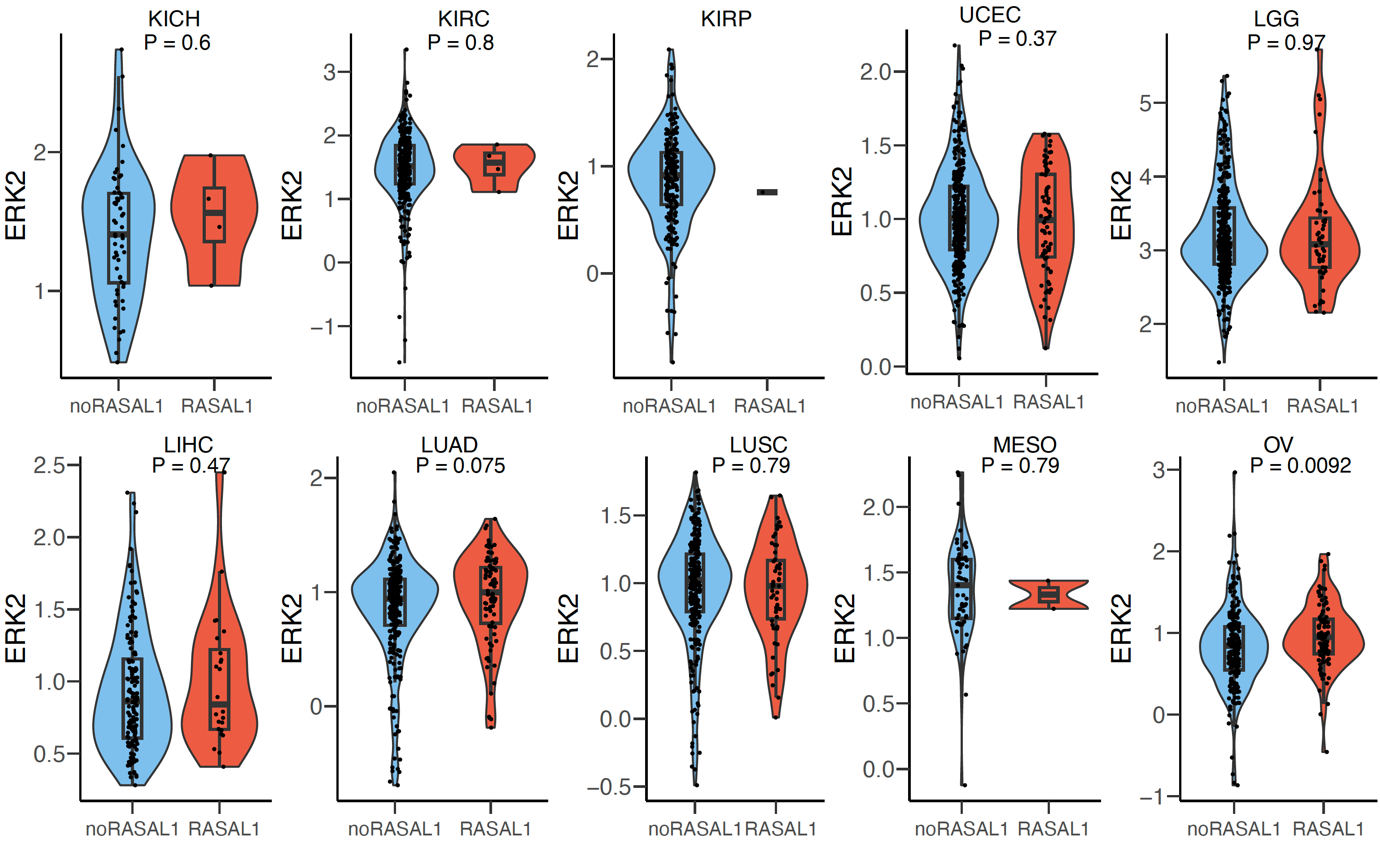


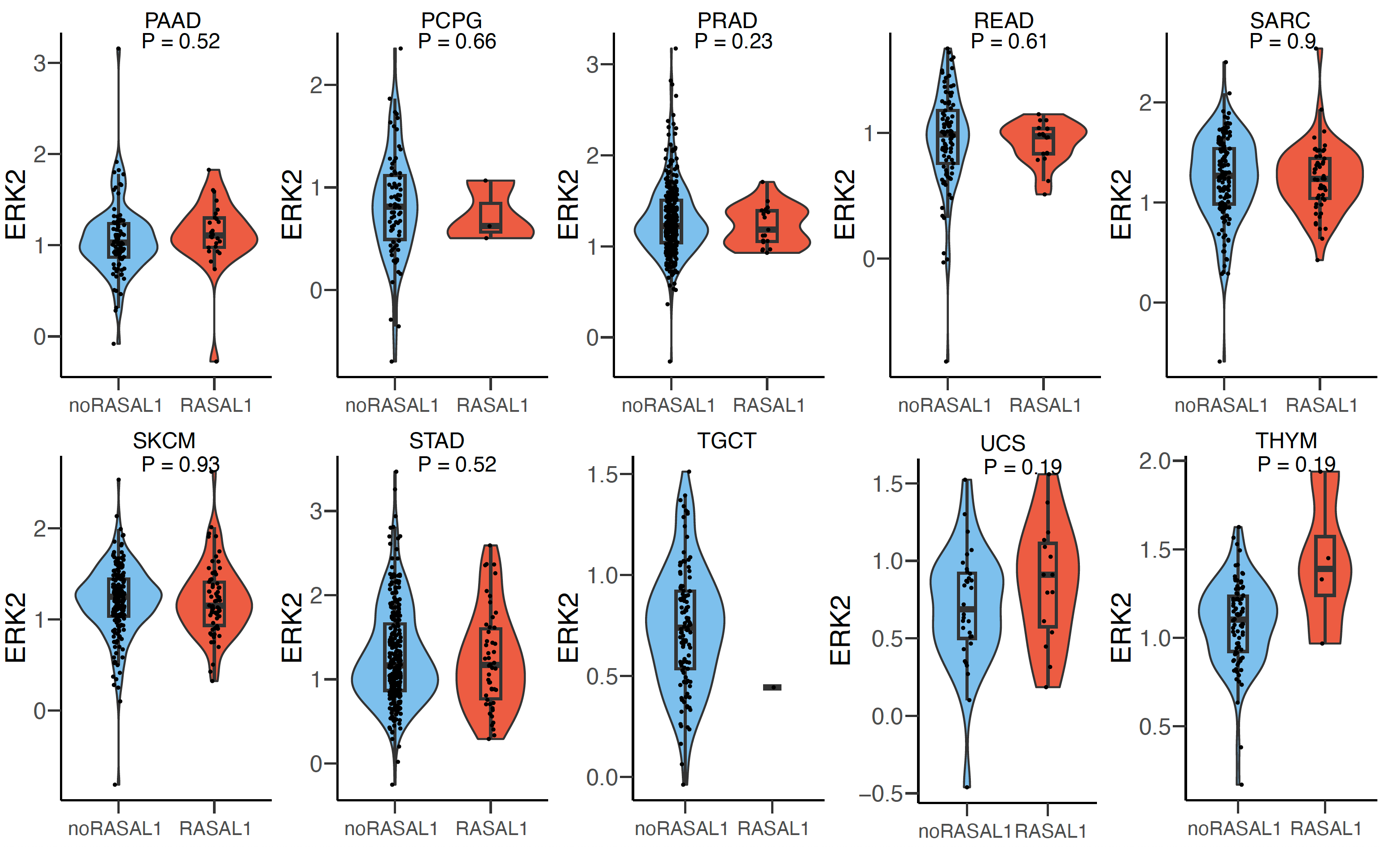


**Figure S5.** **The relationship between ERK2 protein expression and *RASAL1* alterations**. Shown are the relationship between ERK2 protein expression and *RASAL1* alterations in all cancers collectively and in the 30 different cancer types individually as indicated. noRASAL1, cases with no *RASAL1* alterations; RASAL1, cases with *RASAL1* alterations. The abbreviations of the cancer names are as defined in Figure 1 and Table S1.

**
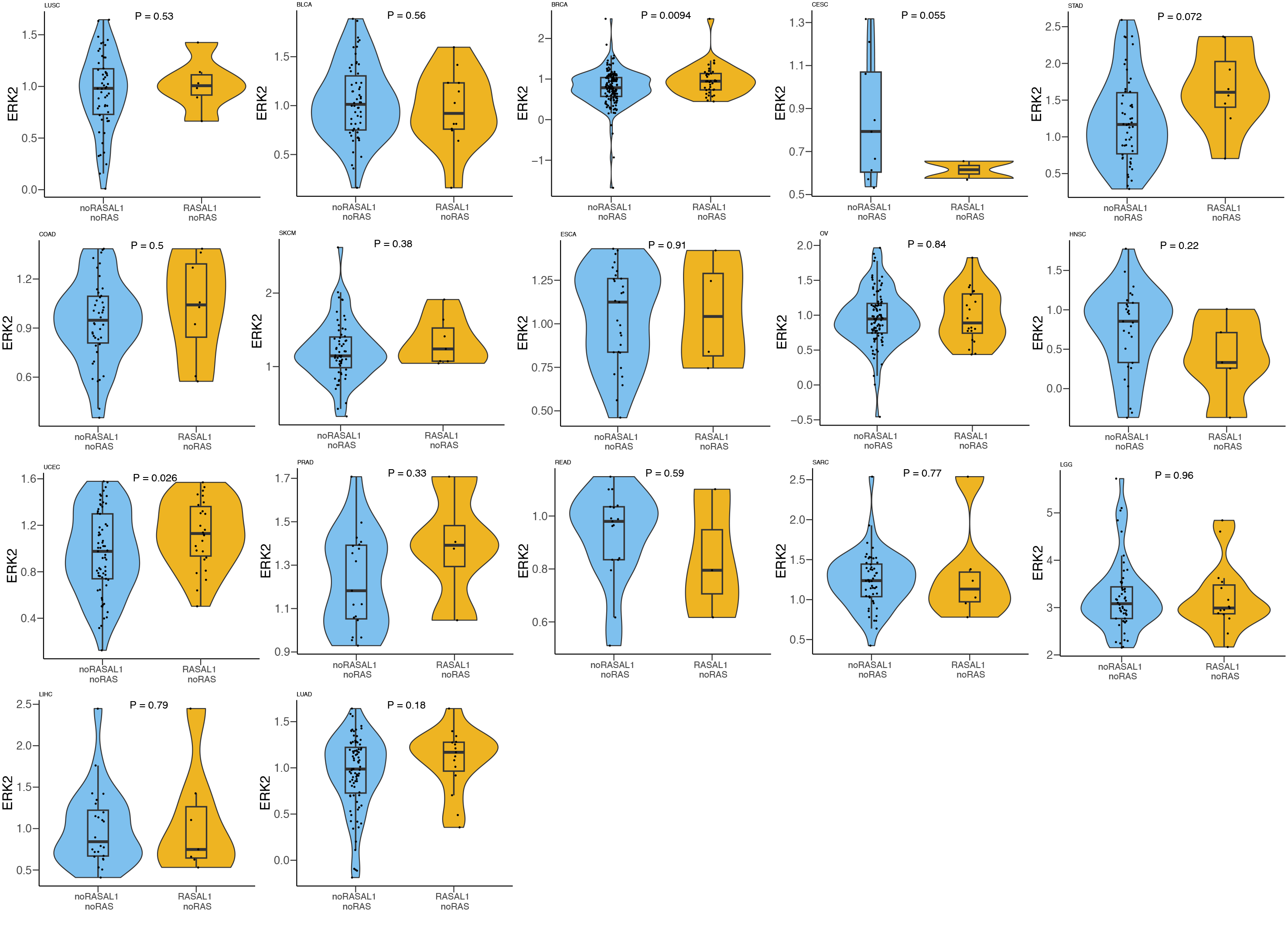
**

**Figure S6.** **The relationship between *RASAL1* alterations and ERK2 protein expression in the absence of *RAS* gene mutations**. Shown are the relationship between ERK2 protein expression and *RASAL1* alterations in all cancers collectively and in the 17 different cancer types individually as indicated. Cancer types with less than 3 samples of *RASAL1* alterations after removal of the *RAS* mutation are not shown here.

noRASAL1noRAS, cases with no *RASAL1* and *RAS* alterations; RASAL1_noRAS, cases with *RASAL1* alterations but no *RAS* alterations. The abbreviations of the cancer names are as defined in Figure 1 and Table S1.

**Figure S7. Kaplan-Meier analyses of the effects of *RASAL1* alterations on the survivals of patients with the indicated cancers.** Shown is the accelerated decline in disease-specific or progression-free survival curve in patients with *RASAL1* alterations (“RASAL1”) compared with that without *RASAL1* alterations (“noRASAL1”) in the indicated individual cancer types. The abbreviations of the cancer names are as defined in Figure 1 and Table S1.

**
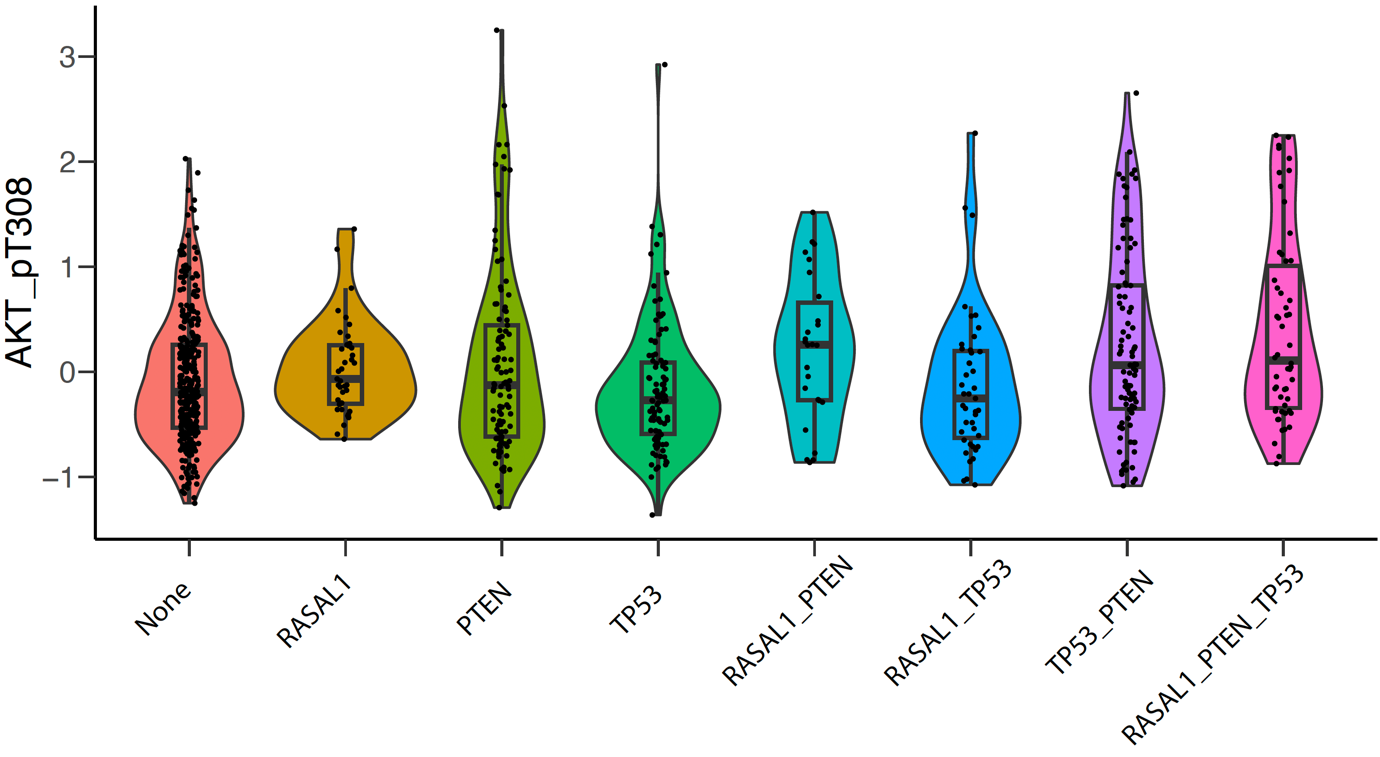

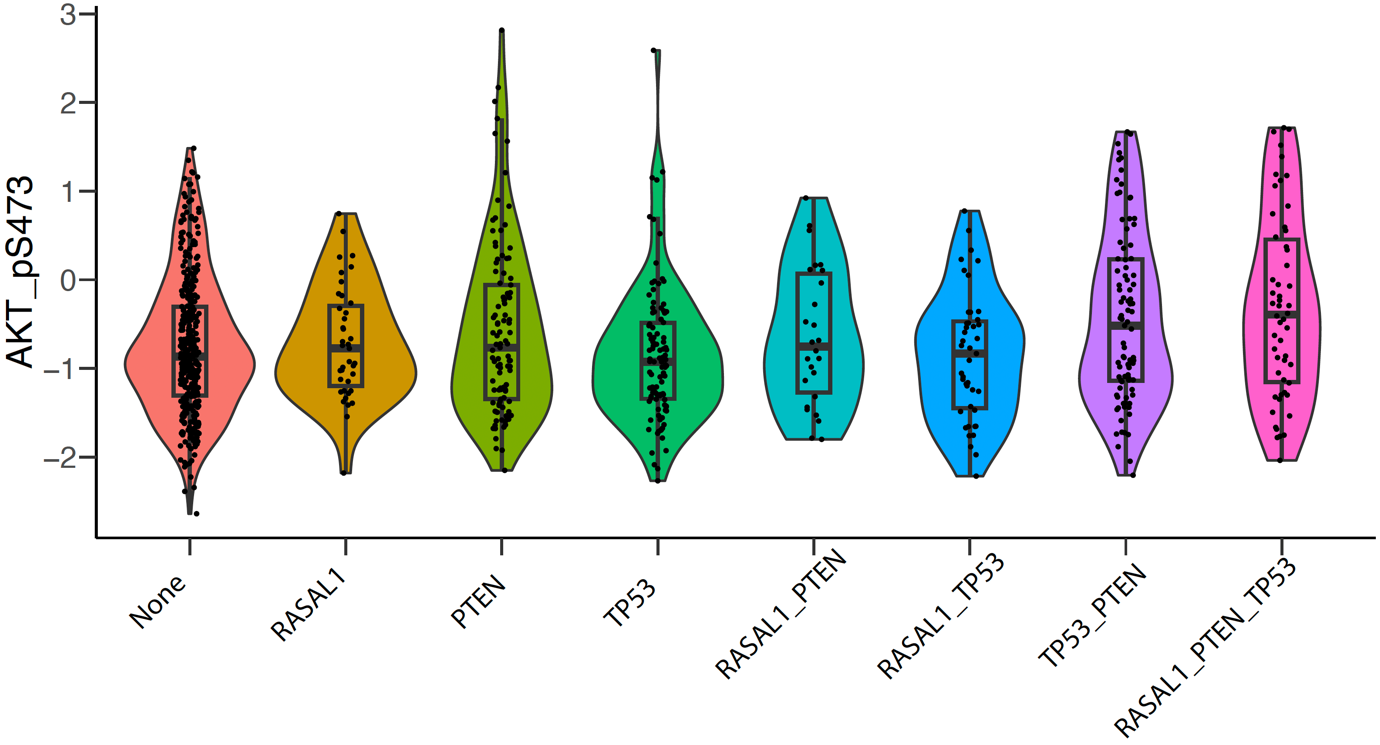
**

**Figure S8.** **Potential cooperative effects of concurrent *RASAL1*, *PTEN* and *TP53* alterations on AKT phosphorylation in BRCA. Comparing with the none alteration group, *RASAL1*, *PTEN* and *TP53* groups have no significantly difference in both phosphorylation levels.**

**Figure S9.** **Successful deletion of *Rasal1* in *Rasal1* KO mice.** Shown are the Sanger sequencing results indicating a 47-bp fragment deletion, resulting in frameshift and premature STOP for *Rasal1*.

| **Table S1:** **Frequency of the genetic alterations of *RASAL1* in 33 cancer types** | | | | | |
| --- | --- | --- | --- | --- | --- |
| **Cancer type** | **Full name** | **# Samples with both mut&copy information** | **# *RASAL1*_mut** | **# *RASAL1*_loss** | **#Total_Mut+Loss** |
| ACC | Adrenocortical carcinoma | 90 | 0(0) | 5(5.56) | 5(5.56) |
| BLCA | Urothelial bladder cancer | 406 | 9(2.22) | 65(16.01) | 73(17.98) |
| BRCA | Breast cancer | 1006 | 6(0.6) | 191(18.99) | 196(19.48) |
| CESC | Cervical cancer | 279 | 4(1.43) | 24(8.6) | 28(10.04) |
| CHOL | Cholangiocarcinoma | 36 | 0(0) | 8(22.22) | 8(22.22) |
| COAD | Colon adenocarcinoma | 399 | 11(2.76) | 54(13.53) | 65(16.29) |
| DLBC | Lymphoid Neoplasm Diffuse Large B-cell Lymphoma | 37 | 1(2.7) | 1(2.7) | 2(5.41) |
| ESCA | Esophageal carcinoma | 184 | 0(0) | 43(23.37) | 43(23.37) |
| GBM | Glioblastoma multiforme | 380 | 3(0.79) | 34(8.95) | 36(9.47) |
| HNSC | Head and neck squamous cell carcinoma | 501 | 4(0.8) | 53(10.58) | 57(11.38) |
| KICH | Chromophobe renal cell carcinoma | 66 | 2(3.03) | 3(4.55) | 4(6.06) |
| KIRC | Clear cell kidney carcinoma | 365 | 1(0.27) | 3(0.82) | 4(1.1) |
| KIRP | Papillary kidney carcinoma | 279 | 1(0.36) | 0(0) | 1(0.36) |
| LAML | Acute Myeloid Leukemia | 133 | 0(0) | 0(0) | 0(0) |
| LGG | Lower Grade Glioma | 509 | 2(0.39) | 61(11.98) | 63(12.38) |
| LIHC | Liver hepatocellular carcinoma | 356 | 3(0.84) | 43(12.08) | 46(12.92) |
| LUAD | Lung adenocarcinoma | 512 | 9(1.76) | 123(24.02) | 131(25.59) |
| LUSC | Lung squamous cell carcinoma | 482 | 17(3.53) | 74(15.35) | 89(18.46) |
| MESO | Mesothelioma | 82 | 1(1.22) | 3(3.66) | 4(4.88) |
| OV | Ovarian serous cystadenocarcinoma | 403 | 5(1.24) | 120(29.78) | 125(31.02) |
| PAAD | Pancreatic adenocarcinoma | 176 | 1(0.57) | 41(23.3) | 42(23.86) |
| PCPG | Pheochromocytoma and Paraganglioma | 162 | 0(0) | 3(1.85) | 3(1.85) |
| PRAD | Prostate adenocarcinoma | 491 | 3(0.61) | 24(4.89) | 27(5.5) |
| READ | rectal adenocarcinoma | 144 | 1(0.69) | 19(13.19) | 20(13.89) |
| SARC | Sarcoma | 234 | 1(0.43) | 58(24.79) | 59(25.21) |
| SKCM | Cutaneous melanoma | 363 | 21(5.79) | 88(24.24) | 98(27) |
| STAD | Stomach adenocarcinoma | 437 | 11(2.52) | 56(12.81) | 67(15.33) |
| TGCT | Testicular germ cell cancer | 145 | 0(0) | 2(1.38) | 2(1.38) |
| THCA | Papillary thyroid carcinoma | 484 | 0(0) | 0(0) | 0(0) |
| THYM | Thymoma | 123 | 0(0) | 7(5.69) | 7(5.69) |
| UCEC | Uterine corpus endometrial carcinoma | 524 | 34(6.49) | 56(10.69) | 90(17.18) |
| UCS | Uterine carcinosarcoma | 56 | 0(0) | 17(30.36) | 17(30.36) |
| UVM | Uveal Melanoma | 80 | 0(0) | 2(2.5) | 2(2.5) |
| Total | | 9924 | 151 (1.52) | 1281 (12.91) | 1414 (14.25) |

| **Table S2: Co-existences between *RASAL1* alterations and *PTEN* alterations or *TP53* mutations** | | | | |
| --- | --- | --- | --- | --- |
| ***PTEN*** |  |  |  |  |
| RASAL1 genetic alterations (n/N (%)) | | PTEN genetic alterations (n/N (%)) | | Adjusted P_value |
| PTEN- | PTEN+ | RASAL1- | RASAL1+ |  |
| 665/6362 (10.45) | 749/3562 (21.03) | 2814/8510 (33.07) | 749/1414 (52.97) | 4.02E-47 |
| ***TP53*** |  |  |  |  |
| RASAL1 genetic alterations (n/N (%)) | | TP53 mutation (n/N (%)) | | Adjusted P_value |
| TP53- | TP53+ | RASAL1- | RASAL1+ |  |
| 498/6147 (8.10) | 916/3777 (24.25) | 2861/8510 (33.62) | 916/1414 (64.78) | 2.43E-110 |

| **Table S3. Association between genetic alterations of *RASAL1* and those of *PTEN* or *TP53* in individual cancer types** | | | | | |
| --- | --- | --- | --- | --- | --- |
| Cancer type | *RASAL1* genetic alteration (n/N (%)) | | *PTEN* genetic alteration (n/N (%)) | | P-adjust |
|  | *PTEN* without alterations | *PTEN* with alterations | *RASAL1* without alterations | *RASAL1* with alterations |  |
| BRCA | 102/655 (15.57) | 91/308 (29.55) | 217/770 (28.18) | 91/193 (47.15) | 1.81E-05 |
| COAD | 7/118 (5.93) | 13/34 (38.24) | 21/132 (15.91) | 13/20 (65) | 0.000173 |
| PRAD | 5/221 (2.26) | 16/111 (14.41) | 95/311 (30.55) | 16/21 (76.19) | 0.000448 |
| STAD | 18/203 (8.86) | 21/84 (25) | 63/248 (25.40) | 21/39 (53.85) | 0.003609 |
| SARC | 16/108 (14.81) | 45/139 (32.37) | 94/186 (50.54) | 45/61 (73.77) | 0.006521 |
| SKCM | 17/98 (17.3) | 56/180 (31.11) | 124/205 (60.49) | 56/73 (76.71) | 0.043517 |
|  |  |  |  |  |  |
| Cancer type | *RASAL1* genetic alteration (n/N (%)) | | *TP53* genetic alteration (n/N (%)) | | P-adjust |
|  | *TP53* without alterations | *TP53* with alterations | *RASAL1* without alterations | *RASAL1* with alterations |  |
| BRCA | 89/667 (13.34) | 104/296 (35.14) | 192/770 (24.94) | 104/193 (53.89) | 1.22E-12 |
| LGG | 3/137 (2.19) | 34/146 (23.29) | 112/246 (45.53) | 34/37 (91.89) | 4.37E-07 |
| UCEC | 10/174 (5.75) | 20/68 (29.41) | 48/212 (22.64) | 20/30 (66.67) | 2.80E-05 |
| GBM | 4/195 (2.05) | 14/78 (17.95) | 64/255 (25.10) | 14/18 (77.78) | 8.12E-05 |
| PRAD | 14/309 (4.53) | 7/23 (30.43) | 16/311 (5.14) | 7/21 (33.33) | 0.001143 |
| SKCM | 51/231 (22.08) | 22/47 (46.81) | 25/205 (12.20) | 22/73 (30.14) | 0.003535 |
| HNSC | 3/77 (3.90) | 34/202 (16.83) | 168/242 (69.42) | 34/37 (91.89) | 0.009226 |
| KICH | 0/44 (0) | 4/22 (18.18) | 18/62 (29.03) | 4/4 (100) | 0.03933 |
| **Notes: The full names of cancer type abbreviations are shown in Table S1.** | | | | | |

Table S4 P-value matrix of between-group differences in AKT_pT308 phosphorylation based on *RASAL1*,*PTEN* and *TP53* alterations in breast cancer

| AKT_pT308 | None | *RASAL1* | *PTEN* | *TP53* | *RASAL1_PTEN* | *RASAL1_TP53* | *PTEN_TP53* | *RASAL1_PTEN_TP53* |
| --- | --- | --- | --- | --- | --- | --- | --- | --- |
| *None* | 1 | 0.1373 | 0.0851 | 0.2425 | 0.0396 | 0.6951 | 8.4883E-04 | 2.4058E-04 |
| *RASAL1* | NA | 1 | 0.7078 | 0.0368 | 0.2371 | 0.2113 | 0.0641 | 9.7664E-03 |
| *PTEN* | NA | NA | 1 | 0.0240 | 0.3811 | 0.1407 | 0.1656 | 0.0287 |
| *TP53* | NA | NA | NA | 1 | 0.0147 | 0.7654 | 2.3905E-04 | 6.79E-05 |
| *RASAL1_PTEN* | NA | NA | NA | NA | 1 | 0.0505 | 0.8392 | 0.3081 |
| *RASAL1_TP53* | NA | NA | NA | NA | NA | 1 | 8.3074E-03 | 1.3340E-03 |
| *PTEN_TP53* | NA | NA | NA | NA | NA | NA | 1 | 0.3154 |
| *RASAL1_PTEN_TP53* | NA | NA | NA | NA | NA | NA | NA | 1 |

Table S5 P-value matrix of between-group differences in AKT_pS473 phosphorylation based on *RASAL1*,*PTEN* and *TP53* alterations in breast cancer

| AKT_pS473 | None | *RASAL1* | *PTEN* | *TP53* | *RASAL1_PTEN* | *RASAL1_TP53* | *PTEN_TP53* | *RASAL1_PTEN_TP53* |
| --- | --- | --- | --- | --- | --- | --- | --- | --- |
| *None* | 1 | 0.7641 | 0.0979 | 0.2422 | 0.5101 | 0.3538 | 4.3475E-03 | 3.8257E-03 |
| *RASAL1* | NA | 1 | 0.3186 | 0.2921 | 0.6951 | 0.3432 | 0.0501 | 0.0210 |
| *PTEN* | NA | NA | 1 | 0.0258 | 0.7099 | 0.0551 | 0.2994 | 0.1170 |
| *TP53* | NA | NA | NA | 1 | 0.2321 | 0.9529 | 1.1674E-03 | 1.0838E-03 |
| *RASAL1_PTEN* | NA | NA | NA | NA | 1 | 0.2582 | 0.2526 | 0.1103 |
| *RASAL1_TP53* | NA | NA | NA | NA | NA | 1 | 5.1601E-03 | 2.7148E-03 |
| *PTEN_TP53* | NA | NA | NA | NA | NA | NA | 1 | 0.4745 |
| *RASAL1_PTEN_TP53* | NA | NA | NA | NA | NA | NA | NA | 1 |
